# Species Extirpation and Colonization Reveal Mixed Homogenization and Differentiation Across Terrestrial Taxa After One of the World's Largest Tailings Dam Failures

**DOI:** 10.1002/ece3.74199

**Published:** 2026-08-19

**Authors:** Reginaldo A. F. Gusmão, Eduardo H. Barros, Tássia J. Bertotto, Marianne Azevedo‐Silva, Tamilie Carvalho, Rogerio L. Rodrigues, Eduardo B. Segatto, Renan L. Betzel, Gustavo P. Pizziolo, Rafaela G. Gatti, Fernanda J. da Silva, Thiago Gonçalves‐Souza

**Affiliations:** ^1^ Programa de Pós‐Graduação Em Etnobiologia e Conservação da Natureza, Departamento de Biologia Universidade Federal Rural de Pernambuco Recife Pernambuco Brazil; ^2^ Ello Ambiental Consultoria Colatina Espírito Santo Brazil; ^3^ Amplo Engenharia e Gestão de Projetos Belo Horizonte Minas Gerais Brazil; ^4^ Department of Ecology and Evolutionary Biology University of Michigan Ann Arbor Michigan USA; ^5^ Institute for Global Change Biology, School for Environment and Sustainability University of Michigan Ann Arbor Michigan USA

**Keywords:** biodiversity loss, dam collapse, mining, seed dispersal

## Abstract

In 2015, the failure of the Fundão tailings dam released ~45 million m^3^ of liquefied tailings into Brazil's Doce River basin, contaminating ~670 km of waterways to the Atlantic Ocean. However, the biodiversity consequences of this event for terrestrial organisms remain poorly understood, particularly across multiple terrestrial taxonomic groups and through time. We assessed how direct and indirect exposure to mine tailings affected the abundance and diversity of plants, vertebrates (anurans and birds), and invertebrates (ants, bees, beetles, and butterflies) in a 4‐year monitoring period from 2022 to 2025. As reference sites, we selected areas that had not been affected by mine tailings but were located sufficiently close to the impacted sites to allow organisms to move among them. We quantified effects on the number of individuals and species and partitioned spatiotemporal β‐diversity into colonization and extirpation components to evaluate consequences for biotic homogenization and differentiation. Directly impacted sites exhibited stronger reductions in abundance and unstandardized richness than indirectly impacted and reference sites, but responses varied among taxonomic groups and depended on whether change was evaluated using incidence (species identities) or abundance (dominance). Overall, mine tailings effects operated through two nonexclusive pathways: taxon‐specific changes in population size and shifts in community composition driven by altered extirpation and colonization. Across groups, impacted landscapes could simultaneously exhibit differentiation via site‐specific losses and homogenization via dominance shifts. These results show that mine tailings can restructure biodiversity along multiple dimensions, emphasizing the need to evaluate abundance, composition, and turnover processes when assessing impacts and recovery in diverse ecosystems.

## Graphical abstract

Fundão tailings‐dam failure (2015)—study area and impact gradient. Regional maps locate the Rio Doce Basin in southeastern Brazil and the Fundão dam (red cross), with the black rectangle marking the extent of the main map. The main map follows the downstream tailings pathway along the river corridor (plume highlight). The enlarged inset depicts the terrestrial sampling landscape grouped into three impact classes: direct mine tailings deposition (dMUD), indirect impact (iMUD), and reference sites outside the mine tailings footprint (REF). A secondary inset indicates the dam‐proximal reach corresponding to the enlarged view.

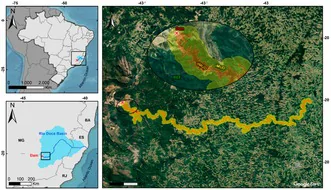

## 1 Introduction

The expansion of extractive capitalism and the continuous demand for development have kept the need for mineral resources at high levels; however, while the economic benefits remain profoundly unequal within society, the environmental impact of meeting this demand comes at a steep price that is borne globally. Despite being crucial to humanity, mining is one of the most environmentally disruptive activities (Islam and Murakami 2021). Its impacts stem primarily from vegetation and soil removal, habitat fragmentation, and pollution (Parkhurst et al. 2025), which contribute to biodiversity loss (Jaureguiberry et al. 2022) and pose substantial risks to human health through exposure to toxic elements (Paulelli et al. 2022). It is estimated that only 1%–3% of each ton of mined material is retained for further processing, with the remainder becoming waste (Adiansyah et al. 2015), primarily stored in tailings dams, with an estimated 29,000–35,000 facilities worldwide (Ma et al. 2026; WMTF 2021). Consequently, tailings dam failures have become a paramount concern within the industry; when these structures fail, the escaping waste can inundate downstream regions orders of magnitude larger than the original mining footprint, particularly when river systems facilitate the long‐range transport and mobilization of these materials (Giljum et al. 2025). From 1915 to 2020, at least 366 tailings dam failures have been reported globally, with environmental damage increasing over the years (Islam and Murakami 2021). In 2015, the Fundão dam in southeastern Brazil collapsed, releasing 45 million m^3^ of liquified tailings into the Doce River basin and contaminating approximately 670 km of waterways to the Atlantic Ocean (Neves et al. 2016). The disaster killed 19 people, left entire communities homeless by completely destroying residential areas and public infrastructure across two districts, and caused losses of US$5.2 billion (do Carmo et al. 2017). The plume affected rivers, tributaries, coastal and marine environments (Aires et al. 2018; do Carmo et al. 2017; dos Reis et al. 2017; Duarte et al. 2021). Fundão is now considered one of the world's worst mining disasters, prompting global reassessments of tailings dam risks and impacts (Islam and Murakami 2021). Yet, the consequences of these catastrophic events for biodiversity remain poorly quantified, especially across multiple taxa and through time.

Large‐scale mining disasters, such as the 2015 Fundão collapse, demonstrate how a single event can cause irreversible damage to biodiversity. The immediate impacts of tailings dam failures include local extinction of aquatic and terrestrial species, increased abundance of antimicrobial resistance genes, habitat destruction, and contamination of crops (Hudson‐Edwards et al. 2024; Kossoff et al. 2014). The Fundão collapse wiped out 470 ha of Atlantic rainforest and 1500 ha of natural reserves, with indigenous lands being severely destroyed (Aires et al. 2018; Omachi et al. 2018). Previous studies show that tailings and associated water contamination can affect biodiversity, but it remains unclear whether terrestrial organisms are consistently negatively impacted and whether their recovery trajectories converge or diverge. For example, aquatic prokaryotic communities changed drastically through time in sites directly affected by mine tailings when compared to water when compared with communities in unaffected waters (Giongo et al. 2020), and the number of fish species was low in mine‐impacted areas (Fráguas et al. 2025). However, species richness and composition of insects did not differ between impacted and nonimpacted sites (Neves et al. 2026). These results highlight the complexity of ecological responses to mining disasters, underscoring the critical need to evaluate multiple taxa to fully understand the extent of environmental degradation and the distinct trajectories of recovery.

Despite actions to promote biodiversity and ecosystem restoration through revegetation (Hudson‐Edwards et al. 2024), predicting biodiversity recovery after tailings dam failures remains challenging, given that their effects can last for years or even centuries (Hudson‐Edwards et al. 2024; Lefcort et al. 2010). This makes long‐term monitoring essential to track population and community responses through time, yet such assessments are still rare and often lack site‐specific, time‐resolved information (Aska et al. 2025; Islam and Murakami 2021). The Fundão disaster represents a critical opportunity to address this gap: active reforestation was implemented after the 2015 collapse, but temporal evaluations of recovery across plant and animal communities remain limited (Campanharo et al. 2020; Garcia et al. 2025). Because the Brumadinho dam collapse occurred only 4 years later (Mello et al. 2024), understanding Fundão's post‐disaster trajectory can directly inform current and future mitigation strategies. More broadly, evaluating biodiversity change through time after Fundão helps fill a global knowledge gap on ecosystem recovery following large‐scale human‐driven catastrophes. Additionally, understanding recovery capacity and environmental costs has substantial legal significance by informing assessments of damage and liability, as well as the development of preventive policies for environmental disasters involving corporate actors.

Here, we investigate the direct impacts on and potential recovery patterns of multiple terrestrial taxonomic groups following the 2015 Fundão collapse, one of Brazil's largest tailings dam failures. We tested how mine tailings affected invertebrates (ants, bees, beetles, and butterflies), vertebrates (anurans and birds), and plants, and specifically addressed the following questions: (1) What is the impact of mine tailings on the abundance and species richness of terrestrial animals and plants, and how does it change over time? We expected mine tailings to reduce abundance and species richness, with these effects weakening over time as habitats and populations recover; (2) Do responses to mine tailings differ in magnitude and direction among terrestrial taxonomic groups? We expected responses to differ among taxonomic groups because of differences in their dependence on the altered substrate and vegetation structure and in their dispersal capacity; (3) What is the effect of mine tailings on colonization and extirpation, and how does it affect β‐diversity homogenization and differentiation over time? We expected extirpation to exceed colonization following the disturbance, with colonization becoming increasingly important during recovery. We also considered two alternative expectations for community composition: communities could become more homogeneous (low β‐diversity) if mine tailings imposed a common environmental filter that favored the same species across sites, or more differentiated (high β‐diversity) if species loss and recolonization varied among sites.

## 2 Materials and Methods

### 2.1 Study Area and Environmental Disaster

The study area includes several regions of the Doce River basin, spanning from the states of Minas Gerais to Espírito Santo, southeastern Brazil. This basin is one of the major river systems in eastern Brazil, extending approximately 888 km from its headwaters to its mouth in the Atlantic Ocean (Pires et al. 2017). The basin is predominantly covered by Atlantic Forest vegetation, a tropical rainforest biome and global biodiversity hotspot characterized by high levels of endemism, with approximately 2% of the area classified as Cerrado, a tropical savanna biome.

The basin has experienced intense historical land use change, resulting in the loss of approximately 71% of its native vegetation (Pires et al. 2017). Current land use is dominated by agriculture, pasture, silviculture, mining activities, and urban areas, and previous estimates indicate that approximately 3.7 million people live within the basin (Pires et al. 2017; PIRH 2010). The regional climate is warm and seasonal, with mean annual temperatures ranging from 18°C to 25°C and annual rainfall between 150 and 1300 mm (Pires et al. 2017).

In November 2015, the Doce River basin became the epicenter of one of the largest mining‐related environmental disasters worldwide. The collapse of the Fundão iron‐ore tailings dam, owned by the mining company Samarco and located in the municipality of Mariana, released approximately 62 million m^3^ of iron‐ore mine tailings into the river system (Neves et al. 2016). The tailings traveled along approximately 600 km of the river channel, reaching the Atlantic Ocean and affecting both aquatic and terrestrial ecosystems along the basin (Escobar 2015; Neves et al. 2016).

The disaster impacted an estimated 2000 ha, affected more than 100 forest fragments, and resulted in the suppression of around 300 ha of native vegetation, including protected areas and private lands (do Carmo et al. 2017). In many locations, vegetation was completely removed following the disaster, either by direct burial under mine tailings or by post‐disaster management actions. The longitudinal spread of mine tailings along the river created a pronounced spatial gradient of disturbance, providing a large‐scale natural experiment to investigate ecological impacts, recovery trajectories, and the effectiveness of restoration strategies across multiple taxonomic groups. Large tropical river basins subjected to mining activities and tailings dam failures are increasingly common worldwide, making the Doce River basin a globally relevant system for understanding biodiversity responses to large‐scale industrial disturbances (do Carmo et al. 2017; Islam and Murakami 2021).

### 2.2 Study Design and Impact Classification

**FIGURE 1:**
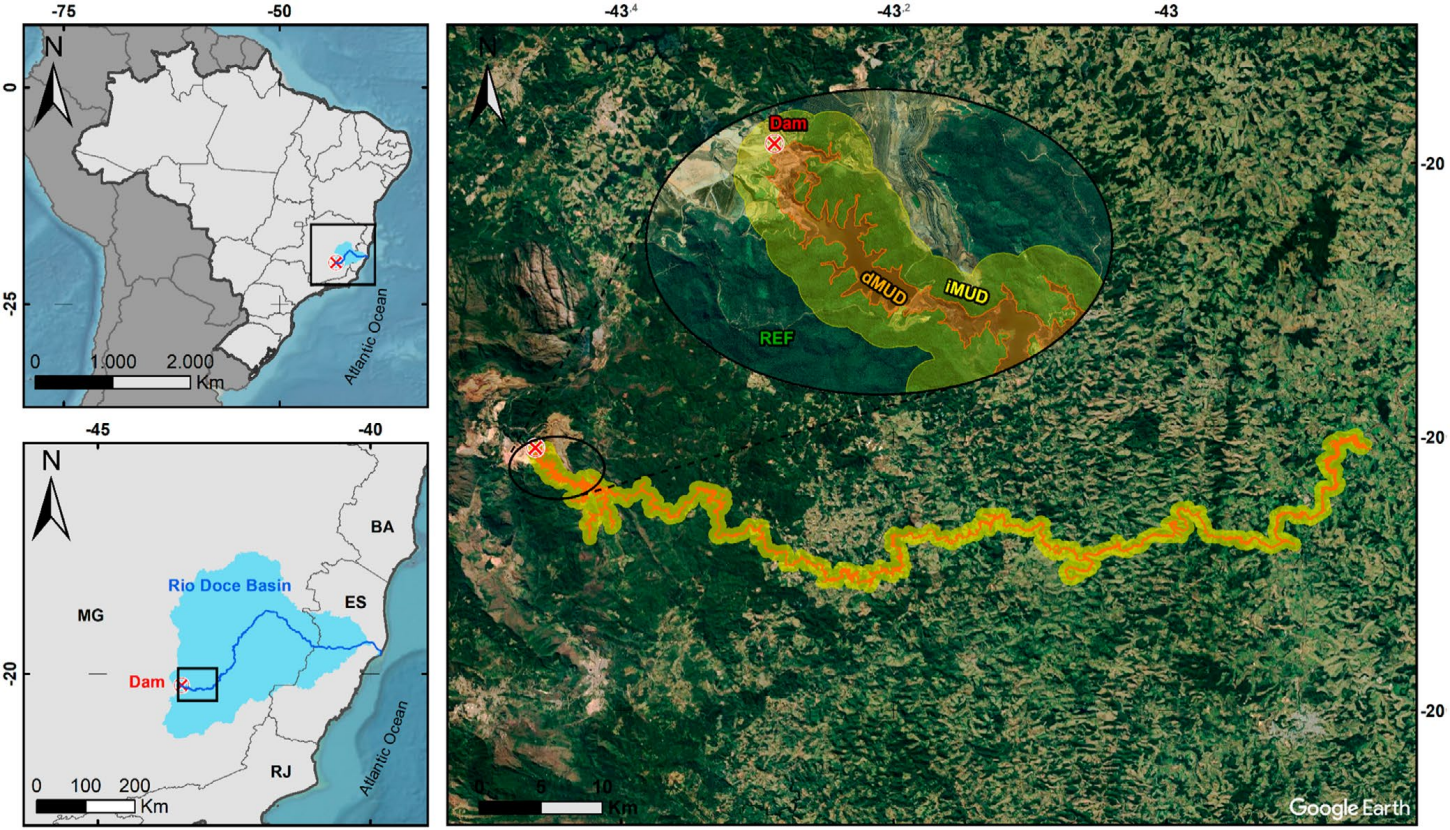
Study area and mapped impact gradient from the 2015 Fundão tailings‐dam failure (Rio Doce Basin, southeastern Brazil). Left panels show the regional context and the Rio Doce Basin, with the Fundão dam location marked by the red crossed symbol and the black rectangle indicating the extent of the main map. The main panel traces the downstream tailings pathway along the river corridor (highlighted plume). The enlarged callout (upper ellipse) shows the terrestrial sampling landscape classified into three impact categories: Areas directly covered by tailings mud (dMUD), indirectly impacted areas (iMUD), and reference areas outside the mud footprint (REF). The smaller ellipse marks the dam‐proximal reach corresponding to the enlarged view.

To capture variation in disturbance intensity and recovery, most sampling sites were established along the main channel of the Fundão tailings dam and the Risoleta Neves hydroelectric power plant, covering approximately 660 km of the river (Figure 1). The Risoleta Neves region is the main affected area, with around 70% of the region still covered with mud (LACTEC 2020). These areas were sampled between 2022 and 2025, totaling 2026 sampling points (123 ± 59 sites per campaign).

Sampling locations were selected based on two main landscape attributes: (i) horizontal distance from the mine tailings and (ii) slope relative to the mine tailings deposition area, using the mine tailings as a reference point (Supporting Information 1—Tables [Link], [Link]). Based on these criteria, sites were classified along a gradient of impact intensity into three categories:

1. Reference sites (hereafter REF) consisted of forest fragments, streams, and lakes located at an average distance of 1769 m from the mine tailings (range: 139–4621 m) and at higher elevations relative to the tailings‐affected areas (elevational difference: 6–290 m). These sites were not directly affected by flooding or burial and therefore provide the best available approximation of reference conditions within the watershed.
2. Indirect mud impact sites (iMUD)—forest fragments or streams and lakes located at an average distance of 728 m (range = 36–3568 m) from the mine tailings but not directly flooded or covered by them and located at a positive slope relative to them (range = 15–146 m). These sites were considered indirectly impacted due to their proximity to the mine tailings, with potential exposure through dust deposition, subsurface flow, and biological vectors such as animal‐mediated transport and trophic transfer.
3. Direct mud impact sites (dMUD)—areas either directly covered by iron‐ore mine tailings or not covered by the mine tailings but flooded by the river during the rainy season. These areas include forest fragments or streams and lakes. Vegetation in these areas was removed after the disaster. We sampled organisms at 24 (±6), 25 (±5), and 74 (±49) locations in the REF, iMUD, and dMUD treatments, respectively (see Table S4).

We acknowledge that using spatial comparisons to evaluate the impacts of tailings‐dam failures and infer potential recovery requires caution, because estimates of species abundance and diversity may also be influenced by factors, such as landscape structure and land‐use history (Aska et al. 2025). However, it is not feasible to establish an experiment in anticipation of a catastrophic environmental disturbance such as a tailings‐dam failure. The occurrence and magnitude of such events are difficult to predict, making assessments of their ecological impacts and subsequent recovery particularly challenging (Hudson‐Edwards et al. 2024). In the absence of pre‐disturbance biodiversity data, unaffected sites within the same watershed provide the best available reference against which to evaluate ecological differences associated with mine‐tailings deposition. Nevertheless, causal interpretations must remain cautious because differences between impacted and reference sites may partly reflect pre‐existing environmental variation. Despite this limitation, our study represents the first large‐scale, multi‐taxon assessment conducted across the affected region and provides important evidence on ecological responses to the disaster, while establishing a foundation for future biodiversity monitoring, restoration, and management in mining‐dominated landscapes.

### 2.3 General and Taxon‐Specific Sampling Protocols

We sampled different taxonomic groups from 2022 to 2025, starting 6 years after the dam collapse, with sampling frequency varying among groups according to their ecology and detectability. Birds were sampled twice per year in the dry and rainy seasons, while anurans, invertebrates and plants were sampled once per year in the rainy seasons. The higher sampling frequency for vertebrates was adopted to improve detection of low abundance groups such as birds to better capture temporal variation in α‐ and β‐diversity. On the other hand, annual sampling during the rainy season tends to yield higher detectability for anurans and invertebrates, and allows for better identification of plants due to the presence of elements such as fruits. Hereafter, we refer to each year or season as a sampling period.

Two 200 × 1 m transects were established in the REF and iMUD treatments, with the first located 10 m from the forest edge and the second at 110 m. Because dMUD sites represented areas where the vegetation was completely removed by the mud, we sampled organisms in 30 × 30 m plots instead of using transects. This approach was necessary because it allowed clearer delineation of sampling boundaries in muddy areas. We accounted for these different methods in two ways. First, we added 30 × 30 m plots in the REF treatment. Second, we compared the effects of transect and plot area on species richness to assess whether differences in sampled area affected our ability to estimate diversity. Below, we describe taxon‐specific protocols to account for differences among taxonomic groups. All taxonomic groups were sampled across the three impact levels (REF, iMUD, and dMUD), except where explicitly stated otherwise. Sampling methods differed among treatments according to habitat structure and the type of sampling unit (transects vs. plots, see Table S4).

#### 2.3.1 Invertebrates

##### 2.3.1.1 Ants and Dung Beetles

Understanding the impacts on ant and beetle communities is relevant due to the ecological role these groups play in ecosystem functioning through seed dispersal and as ecosystem engineers (Griffiths et al. 2015; Dantas et al. 2024). Moreover, these groups are sensitive to substrate structure, moisture, and canopy openness (Inkotte et al. 2022). We sampled epigeic ants using 1‐L pitfall traps (with sticked plastic roof) filled with water and neutral liquid dish soap, providing a standardized sampling protocol for epigeic fauna, excluding strictly arboreal and hypogeal groups. In transect‐based sampling units (REF and iMUD), we established five traps every 50 m, with 480 h of sampling effort per year (10 traps × 48 h; Supporting Information 2: Figure S1). In plot‐based sampling units (REF and dMUD), we installed five pitfall traps per 30 × 30 m plot at each vertex and one in the center, totaling 240 h of sampling effort per year (5 traps × 48 h; see additional details in Supporting Information 2: Figure S2). We sampled dung beetles (Scarabidae) using the same protocol, except that pitfall traps were baited with 50 g of feces to attract them and no central trap was used.

##### 2.3.1.2 Bees

By assessing the impacts on bee communities, we can understand potential changes in ecological interactions such as pollination; furthermore, this group serves as a direct indicator of local floral diversity and plant reproductive phenology (Potts et al. 2003). We sampled bees using pan traps, which use visual attraction and tend to predominantly capture opportunistic and generalist bees (Roulston et al. 2007). We combined this approach with aromatic baits that act as a chemical attractor capturing mainly specific floral‐visiting guilds. By combining these two approaches, we minimized the specialized bias of each standalone technique, providing a more comprehensive multi‐guild assessment of the local bee fauna. First, three containers were colored in fluorescent colors white, yellow, and blue. Second, we used 2‐L plastic bottles with seven aromatic fragrances: Benzyl acetate, Methyl cinnamate, Cineole or Eucalyptol, Eugenol, Vanillin, Methyl salicylate, and Beta‐ionone (Nemésio and Santos Junior 2014). We installed five pan traps and three aromatic traps every 50 m in transect‐based sampling units (REF and iMUD; Supporting Information 2: Figure S1). We added four pan traps at the corners and one aromatic trap in the center of 30 × 30 m plots in the REF and dMUD treatments (Supporting Information 2: Figure S2). We checked all traps every 24 h for two consecutive days.

##### 2.3.1.3 Butterflies

This group is a good indicator of microhabitat quality, being sensitive to changes in temperature and humidity, as well as fruit availability (Uehara‐Prado and Freitas 2009). We sampled frugivorous butterflies using baited Van Someren‐Rydon traps that provide a standardized protocol that eliminates collector bias (DeVries 1987). We added 10 mL of macerated banana bait fermented for 48 h to each trap and hung them 1 m from the ground. In transect‐based units (REF and iMUD), we installed five traps every 50 m along each transect (Supporting Information 2: Figure S4). In plot‐based units (REF and dMUD), we established four traps per 30 × 30 m plot (Supporting Information 2: Figure S1). We maintained traps in the field for three consecutive days with daily checks.

#### 2.3.2 Vertebrates

##### 2.3.2.1 Anurans

This group is sensitive to soil and water contamination; moreover, anurans are top predators of invertebrates and a main link between aquatic and terrestrial ecosystems (Relyea 2005; Whiles et al. 2006). We sampled adult anurans using active sampling in REF, iMUD, and dMUD treatments. Sampling was conducted by searching for individuals in terrestrial and aquatic environments (rivers, streams, lakes, and ponds) during two periods: daytime (9:00 a.m. to 4:00 p.m.) and nighttime (7:00 p.m. to 12:00 a.m.), across four 50 × 5 m transects (Supporting Information 2: Figure S3). Thus, we used a standardized daytime and nighttime active sampling to maximize the detectability of the local anuran fauna across diverse microhabitats. Each transect was surveyed for 30 min per period, and adult anurans were collected by active capture or recorded by auditory detection.

##### 2.3.2.2 Birds

We sought to understand the impacts on bird communities in general and on the specialized pollinator community because these groups respond quickly to changes in forest structure and resources (e.g., fruit and nectar). Moreover, birds play a key role to various ecosystem functions such as seed dispersal, pollination, and predation (Sekercioglu 2006; Morante‐Filho and Faria 2017). Thus, we used standardized censuses to sample birds in all treatments using point counts and artificial feeders. Four‐point counts (two per transect) were established every 200 m at each sampling location in the REF, iMUD, and dMUD treatments. At each point count, we recorded all birds detected visually or aurally for 10 min, within a maximum radius of 50 m.

We also used artificial feeders to attract specific bird groups, such as hummingbirds. Feeders (*n* = 2 per transect) were installed on different days to avoid overlap with the point count surveys. The feeders were placed at a height of 1.5 m and spaced 5 m apart. Before quantifying bird visits, feeders were left in place for two to three days to allow birds to habituate (Kormann et al. 2016) (Supporting Information 2: Figure S4). We then conducted censuses by recording all birds visiting the feeders for 25 min, from sunrise until 9:30 a.m. for point count and until 5:00 p.m. to feeders, with two repetitions on different days for each feeder.

#### 2.3.3 Plants

As primary producers, this group forms the foundation of the ecosystem and was physically removed from the areas directly affected (dMUD) by the dam collapse (Aires et al. 2018; Omachi et al. 2018). Additionally, plants are sensitive to physical soil alterations and water stress caused by the deposition of mining tailings (Schippers et al. 2000; Mendez and Maier 2008). We sampled plants in 30 × 30 m plots across all treatments. We installed three plots per transect in the REF and iMUD treatments and between one and six plots in the dMUD treatment, depending on the area. All individuals with DBH ≥ 15.7 cm were marked (for temporal comparisons) and identified (Supporting Information 2: Figure S3).

### 2.4 Data Analyses

Prior to analysis, we performed taxonomic harmonization to ensure data quality by standardizing inconsistent labels and morphospecies notation and by checking scientific names and synonyms using the R packages *taxize* (Chamberlain and Szöcs 2013) and *rgbif* (Chamberlain et al. 2026).

We used generalized linear mixed models (GLMMs) to test how impact level and sampling period affect the number of individuals (abundance) and the number of species (richness) for each taxonomic group. Impact level and sampling period were included as fixed effects, while plot identity was included as a random effect to account for repeated measurements. Models assumed a negative binomial error distribution with the quadratic mean–variance parameterization (nbinom2 sensu Hardin and Hilbe 2018), which provided a better fit than the Poisson distribution across all taxonomic groups, as indicated by overdispersion diagnostics. This choice is appropriate for our data because we expect variance to increase nonlinearly with the mean (Zuur et al. 2009), for example reflecting higher variability in species richness at sites with larger numbers of individuals sampled.

We also fitted conditional models in which log(abundance) was included as an offset in the GLMM to account for the expectation that sites with more individuals (used here as a proxy for sampling effort) will tend to have higher observed species richness. This approach yields an abundance‐standardized richness metric, in which richness is modeled conditional on abundance rather than assumed to vary independently. Importantly, this standardization represents a statistical adjustment on the log scale and does not imply a strict biological proportionality between species richness and abundance. This framework allows us to disentangle whether the effects of impact level and sampling period on richness arise directly or indirectly through changes in abundance. We avoided rarefaction‐based approaches because many plots contained fewer than three individuals, a condition under which rarefaction becomes unstable and potentially uninformative, particularly in heavily impacted sites with genuinely low abundance. Model adequacy was evaluated using simulation‐based residual diagnostics implemented in the DHARMa package (Hartig 2024). We generated scaled residuals using the *simulateResiduals* function and evaluated residual uniformity, overdispersion or underdispersion. We also tested for residual spatial autocorrelation using the *testSpatialAutocorrelation* function based on the geographic coordinates of the sampling sites (Hartig 2024). We found no evidence of residual spatial autocorrelation in any fitted model. Finally, we applied Levene's test as a complementary check of variance homogeneity across sampling periods. These diagnostic criteria were applied consistently across all taxonomic groups, irrespective of the direction or statistical significance of the estimated effects.

As a complement, we quantified diversity using Hill numbers (effective number of species) to evaluate how treatment differences depend on the relative abundance distribution (Chao et al. 2014; Jost 2007). Hill numbers form a continuum indexed by the order *q*, which controls sensitivity to rare versus dominant species. For *q* = 0, the metric equals species richness, weighting all species equally and therefore reflecting changes primarily driven by rare species. For *q* = 2, the metric emphasizes dominant species, capturing treatment effects associated with changes in dominance and evenness (Chao et al. 2014). By comparing diversity profiles across *q*, we assessed whether treatment differences (dMUD, iMUD, and REF) were driven by changes in the presence of rare species (*q* = 0) or by shifts in the relative abundance of dominant species (*q* = 2). This provides a direct complement to our analyses contrasting unstandardized richness with richness measures that account for variation in abundance.

**FIGURE 2:**
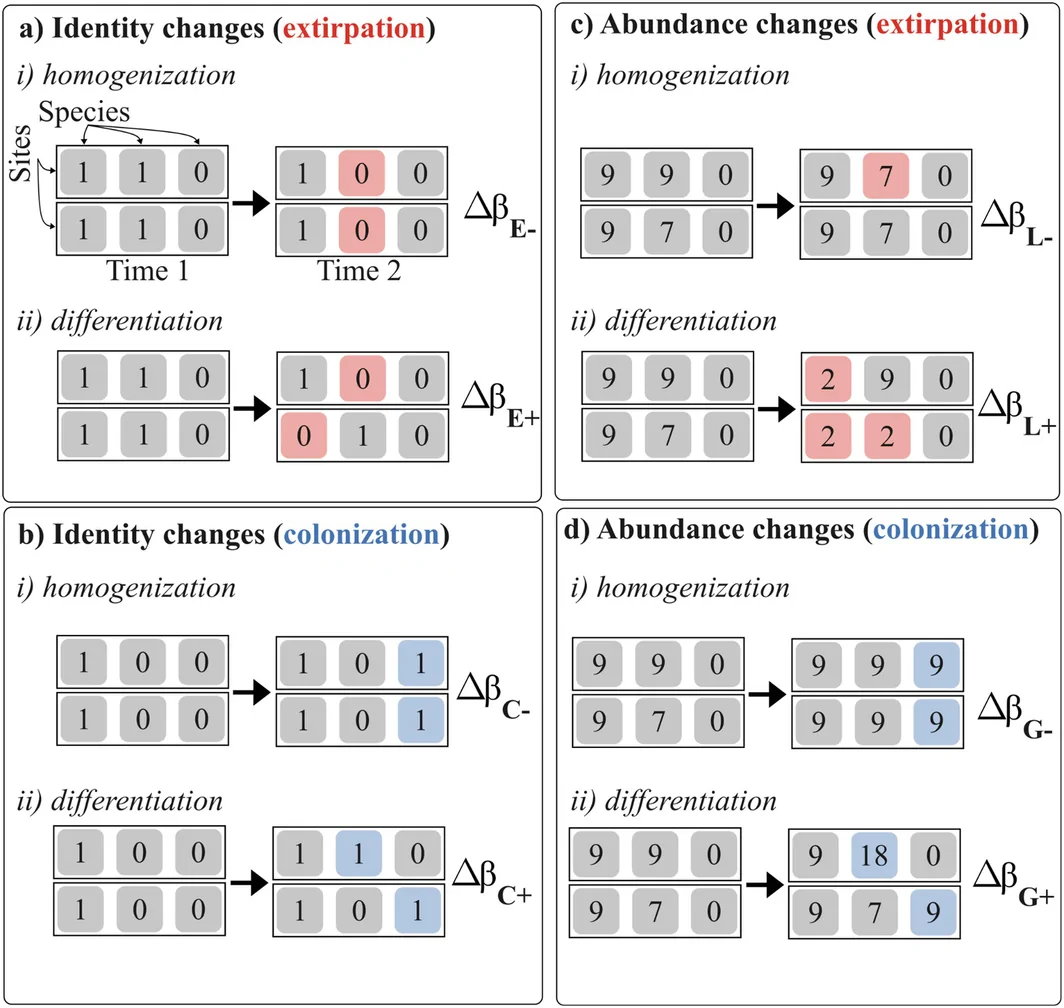
Conceptual illustration of additive partitioning of temporal change in β‐diversity (Δ*β*) into extirpation/loss and colonization/gain components, and their contributions to homogenization versus differentiation. Each panel shows two sites (rows) and three species (columns) at Time 1 and Time 2. Colored cells indicate the species or abundance changes responsible for Δ*β* between time points. (a, b) Identity‐based (incidence) change: Extirpations (red) and colonizations (blue) can produce homogenization (i; “−”) when the same species are lost or gained in both sites, or differentiation (ii; “+”) when losses or gains occur in different species across sites. (c, d) Abundance‐based change: Losses (red) and gains (blue) in local abundances similarly contribute to homogenization (i; “−”) when changes occur in shared species in the same direction across sites, and to differentiation (ii; “+”) when changes are site‐specific (i.e., different species change across sites). The labels E, C, L, and G denote, respectively, the extirpation‐, colonization‐, loss‐, and gain‐resultant components of Δ*β* and their direction toward homogenization (−) or differentiation (+).

We used the additive partitioning framework developed by Tatsumi et al. (2021, 2022) to quantify spatiotemporal changes in species composition (β‐diversity) by disentangling the contributions of colonization and extirpation to biotic homogenization or differentiation (Figure 2). The primary strength of this analysis is its ability to decompose changes in β‐diversity (Δ*β*_total_) into components driven by incidence‐based dynamics (extirpation or colonization of species; Figure 2a,b) and abundance‐based dynamics (quantitative losses or gains in local population sizes; Figure 2c,d). For each pair of sites, the method compares β‐diversity between two sampling periods and determines whether each type of community change made the sites more similar or more dissimilar through time. Thus, it does not simply quantify the number of extirpation or colonization events, but quantifies their contribution to temporal changes in compositional dissimilarity. Negative component values indicate that a process contributed to homogenization, whereas positive values indicate that it contributed to differentiation (Figure 2). We applied the incidence‐ and abundance‐based partitions separately. For incidence data, the total change in Jaccard dissimilarity was partitioned into extirpation and colonization components (Δ*β*total = Δ*β*E + Δ*β*C). For abundance data, the total change in Bray–Curtis dissimilarity was partitioned into abundance‐loss and abundance‐gain components (Δ*β*total = Δ*β*L + Δ*β*G). Thus, the incidence‐based components describe changes in species identity, whereas the abundance‐based components describe changes in quantitative community composition. The analyses were performed with the R package *ecopart* (Tatsumi et al. 2022).

More specifically, we calculated the following components, as defined by Tatsumi et al. (2021, 2022):

- Changes in species identity driven by extirpation (Δ𝛽_E_; Figure 2a): This component measures how the local disappearance of species between time 1 and time 2 affects compositional dissimilarity between sites. Extirpation causes homogenization when species unique to individual sites disappear, thereby reducing differences in species composition between sites (Δ*β*E−). Conversely, extirpation causes differentiation when it reduces the species shared between sites, thereby making their compositions more dissimilar (Δ*β*E+).
- Changes in species identity driven by colonization (Δ𝛽_C_; Figure 2b): This component measures how the arrival of species between time 1 and time 2 affects compositional dissimilarity between sites. Colonization causes homogenization when a species arrives at a site where it was previously absent but was already present at the other site, thereby increasing species sharing (Δ*β*C−). Conversely, colonization causes differentiation when a species colonizes only one of the compared sites and remains absent from the other, thereby increasing compositional differences (Δ*β*C+).
- Changes in species abundance driven by loss of individuals (Δ𝛽_L_; Figure 2c): This component measures how decreases in local species abundances affect quantitative dissimilarity between sites. Abundance losses cause homogenization when they reduce differences in species abundances between sites, such as when the abundance of a species declines at the site where it was initially more abundant (Δ*β*L−). Conversely, abundance losses cause differentiation when they increase quantitative differences between sites, including when losses reduce the abundance component shared by the two communities (Δ*β*L+).
- Changes in species abundance driven by gains of individuals (Δ𝛽_G_; Figure 2d): This component measures how increases in local species abundances affect quantitative dissimilarity between sites. Abundance gains cause homogenization when they reduce differences in species abundances between sites, such as when a species increases at the site where it was initially less abundant (Δ*β*G−). Conversely, abundance gains cause differentiation when they increase quantitative differences, such as when a species increases further at the site where it was already more abundant (Δ*β*G+).

For each taxonomic group, impact, index type (incidence‐ or abundance‐based), and Δ𝛽 component, we summarized the distribution of pairwise Δ𝛽 values across all site pairs and time steps by estimating the average and its 95% confidence interval (CI). CIs were computed as mean ± 1.96 × SE, with SE = SD/√*n*, and *n* as the number of pairwise site comparisons (across all available time steps) for a given taxonomic group, impact category, index type, and Δ𝛽 component.

## 3 Results

### 3.1 Effects of Mine Tailings Impact and Sampling Period on Alpha Diversity

**FIGURE 3:**
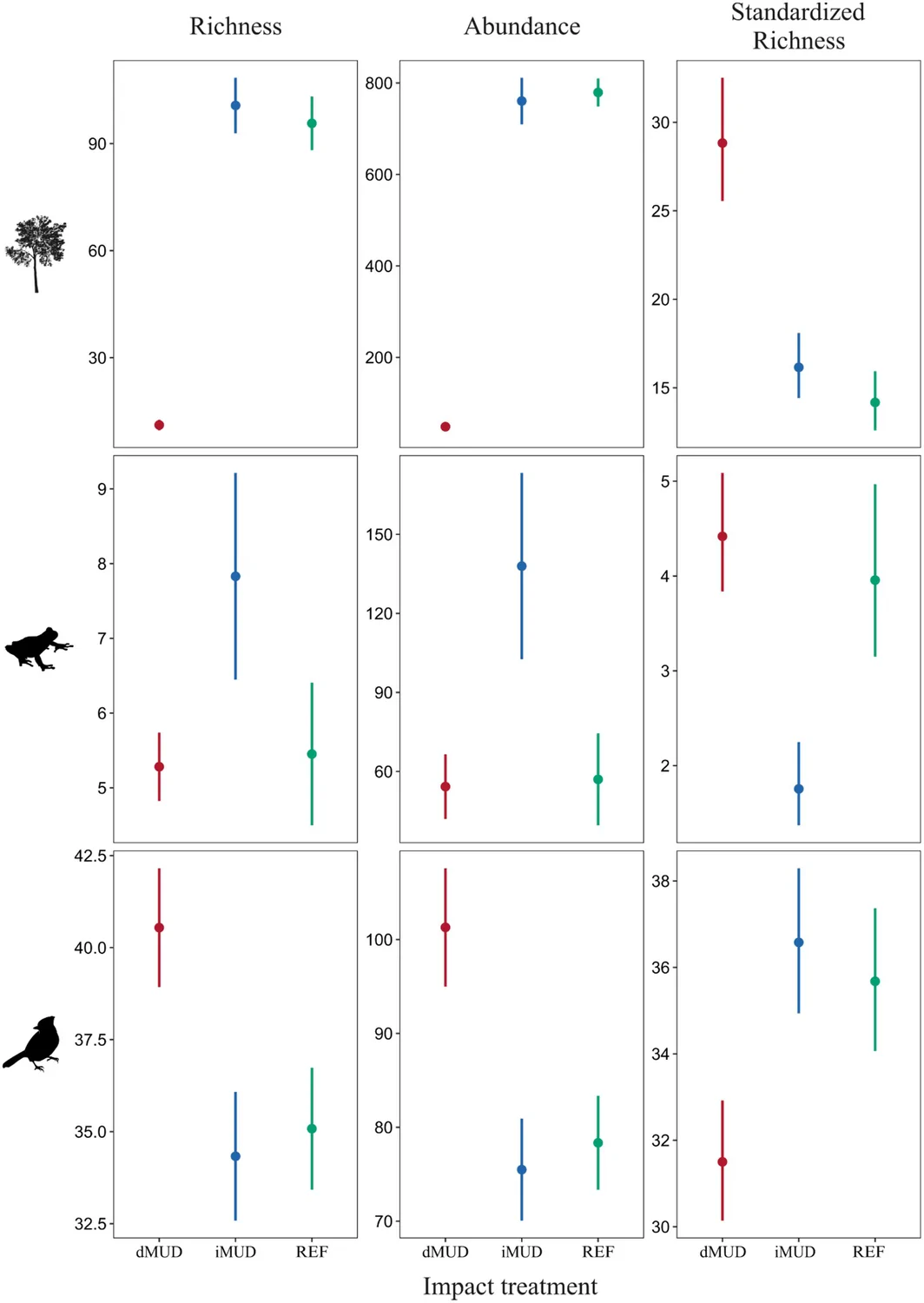
Effects of mud tailings on α‐diversity for plants and vertebrates. Richness, abundance, and abundance‐standardized richness are shown for reference (REF), indirectly impacted (iMUD), and directly impacted (dMUD) sites across sampling periods for plants and vertebrates. Points indicate estimated means and vertical bars show 95% confidence intervals.

We found that the effects of mine tailings on alpha diversity varied strongly among taxonomic groups and operated both directly and indirectly through changes in organism abundance. Using unstandardized, “sampling effort‐free” metrics, we observed a negative association between sites directly impacted by mine tailings and both abundance and species richness of ants, bees, beetles, and plants (Figures 2 and 3, Table 1; see Supporting Information 3 for pairwise contrasts between treatments: Tables S5–S8). These differences are consistent through time (Supporting Information 3, Figures S5 and S6).

**TABLE 1:** Summary of likelihood‐ratio tests from GLMMs evaluating the effects of impact level and sampling period on abundance, richness, and abundance‐standardized richness across taxonomic groups.

Taxonomic group	Factor	df	Abundance		Richness		Abundance‐standardized richness	
			*χ* ^2^	*p*	*χ* ^2^	*p*	*χ* ^2^	*p*
Plants	Impact level	2	425.0	< 0.001	470.5	< 0.001	153.9	< 0.001
	Sampling period	4	0.7	0.954	1.8	0.779	4.7	0.318
Anura	Impact level	2	39.9	< 0.001	21.6	< 0.001	48.6	< 0.001
	Sampling period	3	57.4	< 0.001	55.6	< 0.001	33.1	< 0.001
Birds	Impact level	2	56.7	< 0.001	34.3	< 0.001	37.9	< 0.001
	Sampling period	7	78.8	< 0.001	80.8	< 0.001	137.9	< 0.001
Bees	Impact level	2	239.7	< 0.001	147.0	< 0.001	133.2	< 0.001
	Sampling period	3	73.6	< 0.001	86.9	< 0.001	14.0	0.003
Beetles	Impact level	2	174.2	< 0.001	72.4	< 0.001	36.9	< 0.001
	Sampling period	3	21.9	< 0.001	55.3	< 0.001	59.1	< 0.001
Ants	Impact level	2	57.6	< 0.001	98.6	< 0.001	164.7	< 0.001
	Sampling period	3	40.6	< 0.001	30.7	< 0.001	28.6	< 0.001
Butterflies	Impact level	2	8.7	0.013	15.5	< 0.001	18.3	< 0.001
	Sampling period	3	51.4	< 0.001	50.2	< 0.001	10.5	0.015

**FIGURE 4:**
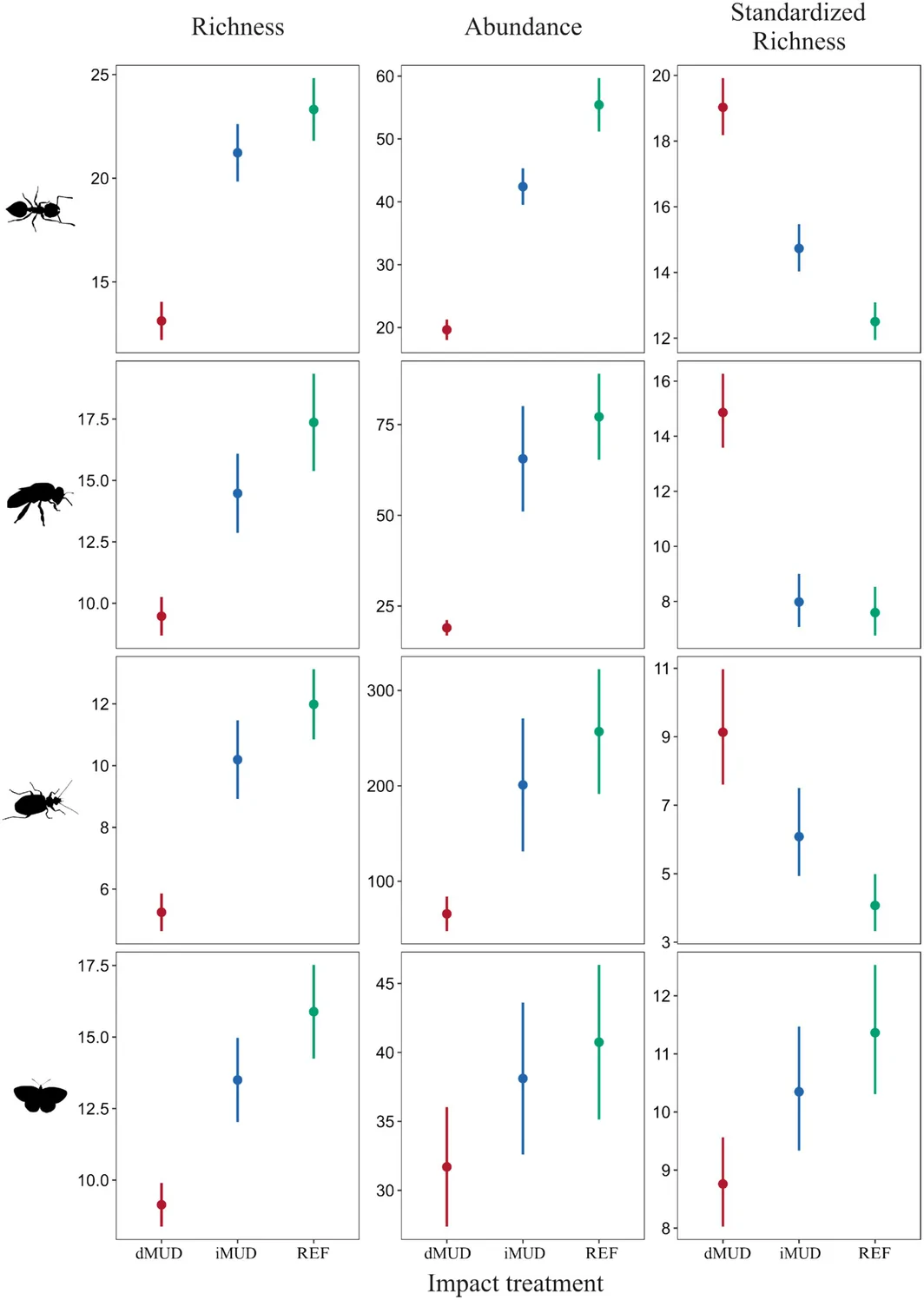
Effects of mud tailings on α‐diversity for invertebrates. Richness, abundance, and abundance‐standardized richness are shown for reference (REF), indirectly impacted (iMUD), and directly impacted (dMUD) sites across sampling periods for ants, bees, beetles, and butterflies. Points indicate estimated means and vertical bars show 95% confidence intervals.

Importantly, these patterns changed when richness was standardized by abundance, thereby accounting for differences in sampling effort and local population size. Abundance‐standardized richness represents the number of species expected for a given number of individuals and allows us to isolate changes in community structure from changes in abundance per se. Comparisons based on abundance‐standardized richness therefore yielded qualitatively different results from those based on unstandardized richness (Figure 4, Supporting Information 3, Figures S5 and S6). Sites directly impacted by mine tailings (dMUD) exhibited higher abundance‐standardized richness of plants, ants, bees, and beetles, despite having lower total abundance. In contrast, dMUD sites supported fewer bird species per individual than iMUD and REF sites once differences in abundance were accounted for. For butterflies and anurans, abundance‐standardized richness did not differ significantly among treatments.

Together, these contrasting responses highlight two distinct consequences of mine tailings disturbance. First, the reversal from negative effects on unstandardized richness to positive effects on abundance‐standardized richness for plants, ants, bees, and beetles suggests that communities in heavily impacted sites are more even (Supporting Information 3, Figures S7 and S8). Conversely, in less impacted sites, higher unstandardized richness for birds (and, to a lesser extent, anurans) appears to be driven primarily by higher abundance, rather than by a more even distribution of individuals across species (Supporting Information 3, Figures S7 and S8). Second, the large reductions in total abundance observed in heavily impacted sites indicate that alpha diversity alone cannot fully capture the ecological consequences of disturbance. Disentangling whether changes in diversity arise from species loss, species gain, or shifts in relative abundances requires explicit examination of colonization and extirpation processes and their contribution to temporal changes in β‐diversity.

### 3.2 Effects of Mine Tailings on β‐Diversity Homogenization and Differentiation Through Colonization and Extirpation

We compared β‐diversity across taxonomic groups by partitioning the contributions of colonization (and gains of individuals) and extirpation (and losses of individuals), which allowed us to evaluate whether mine tailings promote biotic homogenization or differentiation. Interpreting these results is inherently multidimensional because the same impacted landscape can show (i) changes in species identity versus changes in population sizes, and (ii) opposing signals from extirpation/loss versus colonization/gain. Therefore, even when α‐diversity is declining, β‐diversity can simultaneously increase or decrease depending on whether impacted sites lose the same (homogenization) or different species (differentiation), and whether gains occur in a few widespread taxa or in unique taxa across sites.

**FIGURE 5:**
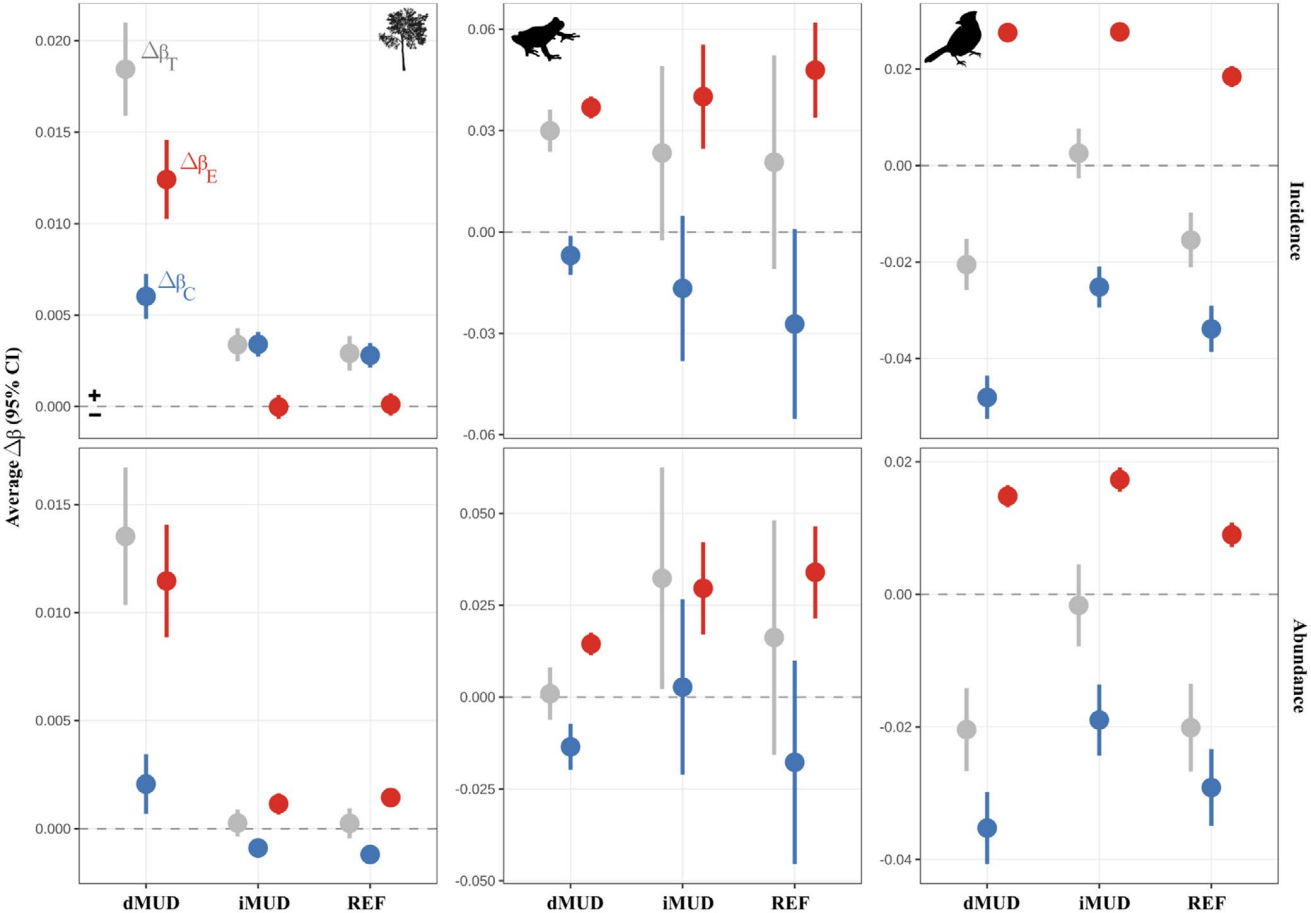
Temporal partitioning of β‐diversity change for plants and vertebrates. Mean pairwise temporal change in β‐diversity (Δ*β*) is shown for reference (REF), indirectly impacted (iMUD), and directly impacted (dMUD) sites, separated by incidence‐based (Jaccard) and abundance‐based (Bray–Curtis) comparisons (rows). Colors indicate the additive partitioning components: Total change (Δ*β*Total), colonization‐/gain‐resultant change (Δ*β*C), and extirpation‐/loss‐resultant change (Δ*β*E). Points show means and vertical bars show 95% confidence intervals based on pairwise site comparisons pooled across time steps.

At the community level (incidence‐based dynamics), extirpation generally increased in dMUD sites and tended to favor differentiation for plants, birds, ants, and beetles relative to iMUD and REF sites (Figures 4 and 5; Table S7). This pattern suggests that direct mine tailings impacts did not simply remove a common subset of shared species across sites; instead, local losses differed among sites, increasing compositional dissimilarity through time. The two main exceptions were anurans and bees, for which extirpation did not increase in mine tailings‐affected areas. For bees, extirpation was associated with homogenization in iMUD and REF sites, implying that broader landscape processes may be filtering bee assemblages and producing more uniform communities even where direct tailings impacts were absent.

Colonization (incidence‐based gains) revealed complementary patterns that help reconcile why α‐ and β‐diversity do not always respond similarly. For ants, birds, beetles, and butterflies, colonization increased in REF sites, indicating that less impacted areas accumulated new species over time. However, the compositional consequence of colonization differed among taxa. First, colonization promoted homogenization for butterflies but differentiation for ants, birds, and beetles, consistent with new species being shared broadly in butterflies but accumulating idiosyncratically across sites in the other groups. Bees showed the opposite spatial pattern, with colonization disproportionately higher in dMUD sites and leading to strong homogenization. This result suggests that a limited subset of bee species colonized or persisted across heavily impacted sites, producing more similar bee communities despite ongoing community turnover.

**FIGURE 6:**
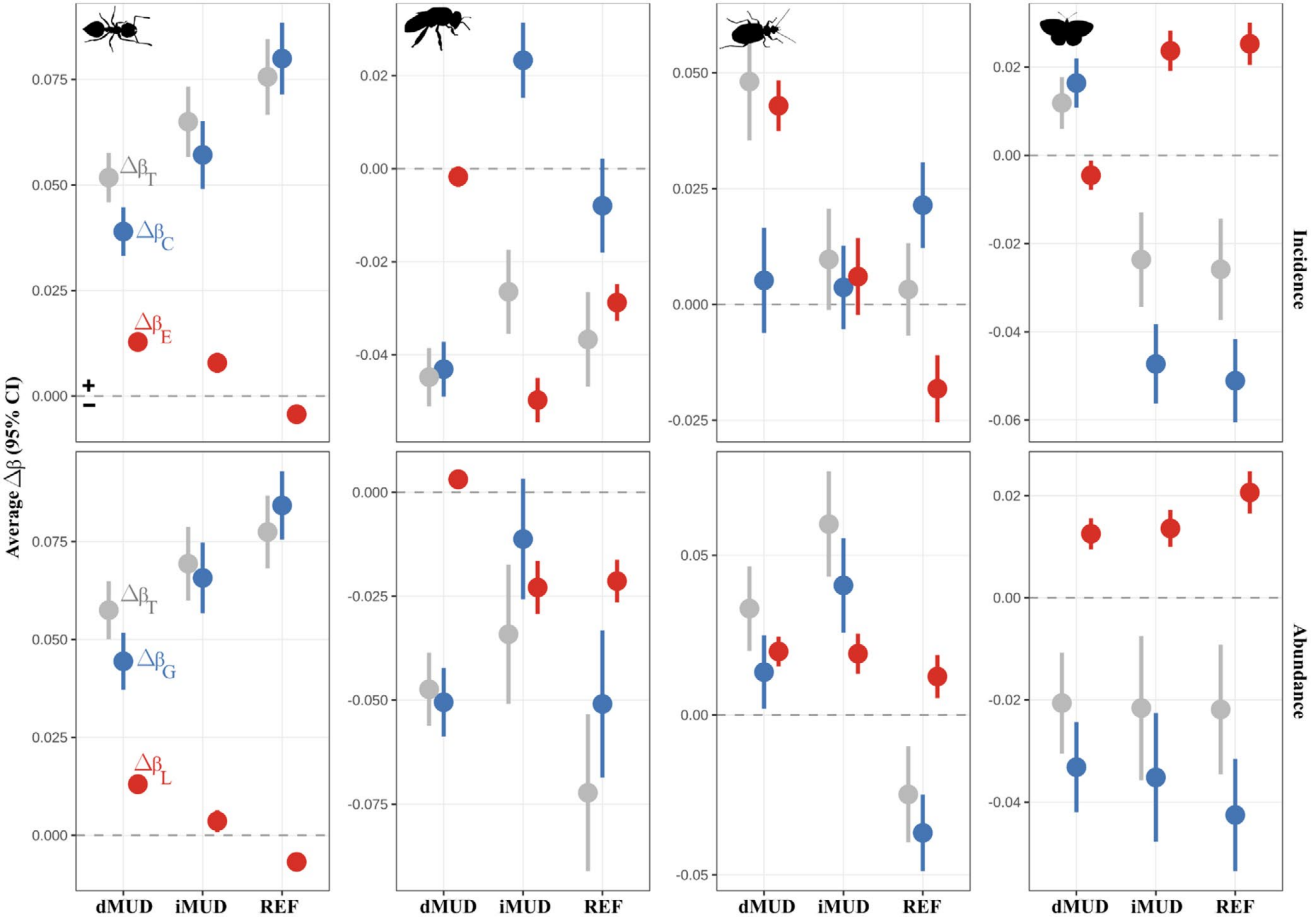
Temporal partitioning of β‐diversity change for invertebrates. Mean pairwise temporal change in β‐diversity (Δ*β*) is shown for reference (REF), indirectly impacted (iMUD), and directly impacted (dMUD) sites for ants, bees, beetles, and butterflies, separated by incidence‐based (Jaccard) and abundance‐based (Bray–Curtis) comparisons (rows). Colors indicate the additive partitioning components: Total change (Δ*β*Total), colonization‐/gain‐resultant change (Δ*β*C), and extirpation‐/loss‐resultant change (Δ*β*E). Points show means and vertical bars show 95% confidence intervals based on pairwise site comparisons pooled across time.

Abundance‐based dynamics (population‐level changes) further demonstrate that identity and dominance can tell different stories about the same disturbance gradient. Sites directly affected by mine tailings exhibited stronger losses of individuals, and these losses were associated with differentiation for ants, bees, birds, and plants (Figures 5 and 6; Table S8); this indicates that population declines were not concentrated in the same species across all sites. In contrast, for anurans, beetles, and butterflies, the magnitude of individual losses and their contribution to differentiation did not differ between mine tailings‐impacted and nonimpacted sites, suggesting either weaker population responses to tailings or stronger temporal variability unrelated to mine tailings exposure.

Patterns of gains in abundance also varied across taxa and helped clarify potential recovery trajectories. For ants, gains were higher in nonimpacted sites than in directly or indirectly affected sites, suggesting that increases in ant abundance were concentrated in REF areas rather than reflecting generalized recovery across the impacted landscape. For birds, abundance gains contributed to homogenization in dMUD sites, indicating that a small number of dominant bird species increased disproportionately near the mine tailings impact, making communities more similar through shifts in dominance. This is especially important for birds because the incidence‐based dynamics suggested differentiation via extirpation. Conversely, although plant gains were also common in dMUD sites, they increased differentiation, implying that different plant species gained in different impacted sites rather than a few species dominating across the entire mud‐affected area. Together, these results show that tailings impacts can simultaneously generate differentiation through site‐specific losses and homogenization through the proliferation of a few dominant taxa, and that the balance between these processes depends on taxonomic group and on whether β‐diversity is viewed through identity‐based versus abundance‐based changes.

## 4 Discussion

### 4.1 General Patterns

Across most taxa, sites directly impacted by mine tailings showed stronger biodiversity loss than indirectly impacted or reference sites, but the magnitude and direction of responses depended on the taxonomic group. Overall, mine tailings effects operated through two nonexclusive pathways: (i) reductions in local abundance (population‐level impacts) and (ii) shifts in species identities via extirpation and colonization (community‐level turnover). Together, these pathways help explain why tailings impacts can generate contrasting signals in α‐ and β‐diversity depending on whether change is evaluated using incidence (species identities) or abundance (dominance). By partitioning spatiotemporal β‐diversity, we further showed that colonization and extirpation can either homogenize communities (when the same species are lost or gained across sites) or differentiate them (when losses or gains are site‐specific). Taken together, these findings indicate that terrestrial communities did not respond uniformly or follow a single recovery trajectory after the 2015 Fundão dam rupture. They also demonstrate that conservation, restoration, and post‐disaster management strategies should not rely solely on single‐taxon indicators or broad, nontargeted recovery strategies.

Consistent with this general picture, directly impacted sites supported fewer individuals and fewer species of ants, bees, beetles, and plants, whereas anurans and birds showed opposite responses. Most insect taxa responded negatively to mine tailings exposure, while indirectly impacted sites did not differ detectably from reference sites, aligning with a previous study in the same region after the Brumadinho tailing disaster, which found no indirect effects on insect communities (Neves et al. 2026). However, by explicitly sampling directly affected areas, we were able to delimit the spatial footprint of tailings impacts on insect communities. Such impacts may also persist for long periods, as mining‐associated effects on insects can last for decades, including reports of slow recovery times (Lefcort et al. 2010). For plants, negative responses are expected because burial and substrate alteration drastically reduced vegetation cover and recruitment, consistent with a ~95% reduction in abundance and richness. For birds and anurans, higher abundance in directly affected sites may reflect greater proximity to riparian habitats and water bodies, which can concentrate foraging and breeding activity (Naiman et al. 1993; Xiang et al. 2017). For example, riparian habitats often attract birds that forage near water (Chan et al. 2008), and many anuran species depend on ponds, lakes, and flooded margins for reproduction (Bateman and Merritt 2020).

Because mine tailings‐impacted sites also yielded fewer individuals, apparent declines in richness can partly reflect reduced encounter rates rather than proportional losses of species. To address this, we estimated abundance‐standardized richness and calculated the effective number of species (Hill numbers), which isolate changes in community structure (evenness and the redistribution of rare species) from changes in total abundance. We found that plants and most invertebrates had higher abundance‐standardized richness in mine tailings‐impacted sites, consistent with more even communities: Total abundance and unstandardized richness decline, but dominance is weakened, so communities appear “richer per individual” when standardized (McCabe and Gotelli 2000). Conversely, the standardized richness of birds was lower in mine tailings‐impacted sites, suggesting a different pattern in which a few dominant bird species disproportionately increase near the mine tailings. This result may be explained by the fact that mine tailings‐impacted sites are restoration areas that tend to favor generalist species. Such dominance can increase competitive asymmetries and contribute to homogenization through shifts in relative abundance, thereby indicating an unbalanced recovery (Holl et al. 2022). It is important to note, however, that higher standardized richness in damaged sites could partly reflect detectability differences or spatial heterogeneity, particularly for visually surveyed taxa where habitat openness can alter encounter rates (Bibby et al. 2000), while also reflecting the ecological reality of these drastically altered environments.

At the community level, mine tailings had contrasting consequences for homogenization and differentiation depending on whether β‐diversity was assessed using species identities or abundances. Incidence‐based partitioning showed that directly impacted sites experienced higher extirpation and increased differentiation for ants, beetles, birds, and plants, suggesting that different species were lost at different sites through time. For these groups, both incidence‐ and abundance‐based partitions tended to indicate differentiation in mine tailings‐impacted sites, reinforcing that losses were not concentrated in the same species across the entire impacted landscape. These patterns are consistent with at least two nonexclusive scenarios. First, because impacts extended over a large area, differentiation may reflect spatially structured recovery, where dispersal limitation and distance‐dependent turnover constrain recolonization and cause sites to lose (and regain) different species over time (e.g., compositional distance decay; Graco‐Roza et al. 2022). This may be particularly relevant for organisms with stronger dispersal limitation, such as some ants, beetles, and plants (Gómez‐Rodríguez and Baselga 2018). Importantly, this pattern also indicates that extirpation continued for nearly a decade after the 2015 rupture. Second, differentiation may also arise from spatial heterogeneity in tailings exposure (e.g., deeper or longer burial and stronger substrate alteration near deposition zones), which can produce site‐contingent losses—particularly in mobile taxa like birds that can actively select less impacted habitats (Tscharntke et al. 2012; Uezu et al. 2005).

Anurans and bees were the main exceptions: species dissimilarity did not differ clearly between impacted and nonimpacted sites. One possibility is that these assemblages were already relatively homogenized regionally, such that pre‐existing drivers (e.g., diseases, habitat loss, and fragmentation) shaped community composition before the tailings disturbance (Mucida et al. 2019; Oswald et al. 2024). In addition, temporal gains suggested potential recovery trajectories in less impacted landscapes. For ants, birds, beetles, and butterflies, colonization increased over time in reference and indirectly impacted sites, reinforcing the role of natural areas as biodiversity reservoirs following large‐scale mining impacts (e.g., Volenec and Dobson 2020).

The impact of this work extends beyond the Doce River basin. Global change is expected to increase the probability of tailings dam failures, for instance through more frequent extreme rainfall events, and such collapses will, in turn, affect biodiversity already threatened by climate change (Aska et al. 2025). This is particularly concerning in biodiversity‐rich regions, where metal mining overlaps with favorable biotic and abiotic conditions sustaining high biodiversity (Parkhurst et al. 2025). By depicting the temporal response of multiple terrestrial taxa following one of the world's largest tailings dam failures, our results underscore that tailings disturbances restructure biodiversity along multiple dimensions—abundance, composition, and dominance—and that these dimensions can move in different directions through time.

### 4.2 Taxon‐Specific Responses

Directly impacted sites were associated with lower richness and abundance of plants due to extirpation of dominant species (e.g., *Syagrus oleracea* and *Piptadenia rigida*), which in turn increased community evenness. Although severely disturbed sites can become dominated by a few stress‐tolerant colonists (i.e., dominance decay—Hillebrand et al. 2008), our results indicate that, in this system, the removal of late‐successional dominants weakened dominance overall and yielded more even assemblages. This result suggests that human‐driven losses of key species may increase community evenness while simultaneously destabilizing ecosystem functioning by reducing functional diversity (Mulder et al. 2004; Hillebrand et al. 2008). Importantly, higher evenness in areas affected by mine tailings should not be interpreted as ecological recovery. For instance, a recent study conducted in the same region showed that ecosystem functioning, including decomposition, remained reduced at directly impacted sites, potentially favoring invasive or disturbance‐adapted taxa such as the invasive grass *Urochloa decumbens* used to feed cattle (Figueiredo et al. 2026). If these taxa respond similarly to the environmental conditions created by severe disturbance, their abundances may become more evenly distributed, thereby increasing community evenness without indicating ecological recovery (see, e.g., Yeboah et al. 2016). We therefore caution against interpreting higher evenness as evidence of recovery in impacted areas, because this metric reflects only the distribution of abundances and does not distinguish species according to their biogeographic origin or functional traits.

Bird communities showed higher abundance and richness in directly impacted areas but lower evenness, consistent with dominance by a small set of disturbance‐tolerant species. This pattern aligns with the winners–losers framework (sensu Tabarelli et al. 2012 and Filgueiras et al. 2021): The loss of sensitive, forest‐dependent specialists (“losers”) is numerically compensated by the proliferation of resilient generalists (“winners”), leading to a functional simplification despite high abundance. This shift toward hyperdominant and uneven communities in environments impacted by anthropogenic activities is well documented in birds across different ecosystems, compromising post‐disturbance community recovery and niche complementarity through the loss of specialized roles (Hillebrand et al. 2008; Filgueiras et al. 2021). In fact, the three most dominant bird species in heavily impacted sites ( *Saltator similis* , *Elaenia flavogaster* , and *Zonotrichia capensis* ) are classified as disturbance‐tolerant generalist species by BirdLife International (2026).

For anurans, mine tailings effects on richness and composition were weak. This muted response may reflect a legacy of historical mining, habitat degradation, and amphibian disease in the region affected by the dam rupture (Carvalho et al. 2017; Pezzuti et al. 2021; Toledo et al. 2023). These factors may have already reduced sensitive taxa and homogenized species pools into more stress‐resistant assemblages. The region affected by the rupture is known as the “Quadrilátero Ferrífero,” due to the high concentrations of metallic elements in the soil, and has a mining history dating back to the 18th century increasing the presence of these metallic elements and others associated with artisanal mining (Costa et al. 2015; Menezes et al. 2020). Consequently, various studies indicate that the region's amphibian community has undergone an evolutionary and historical selection process favoring more resistant species that are capable of surviving in these environments (Reis et al. 2024). Nonetheless, individual‐level impacts of the rupture have been reported and could translate into community‐level change over longer time horizons (Reis et al. 2024). Thus, although we observed a weak effect on amphibians, biological damage at the population level can translate into community‐level alterations through chronic effects (Rohr et al. 2006; Boone et al. 2007; Smalling et al. 2015). Therefore, it is worth noting that the impacts on biological communities resulting from environmental contamination propagate over time, despite being less intense than the direct impact of the dam collapse.

The contrast between birds and anurans supports a trait‐based interpretation: vertebrate responses may be mediated by differences in dispersal ability and habitat requirements (Ficetola et al. 2021; Gusmão et al. 2024). This divergence might be driven by distinct mechanisms during the process of habitat selection (i.e., behavioral and physiological constraints). High mobility allows birds to quickly recolonize altered habitats, often via stress‐tolerant species, because they can actively track landscape‐level resources and overcome degraded matrices to find patches that match their ecological requirements (Sekercioglu 2006; Kennedy et al. 2017). Anurans, however, are constrained by their dependence on aquatic microhabitats and limited dispersal, acting as a physiological barrier to active selection and potentially slowing recolonization due to their permeable skin and reliance on specific aquatic microhabitats (Cushman 2006; Grant et al. 2020). These trait‐dependent responses suggest that mitigation and restoration should adopt a multi‐taxon framework aimed at limiting biotic homogenization and improving connectivity for specialist populations.

We found lower richness and abundance of invertebrates in mine tailing‐affected areas than nonaffected areas, but greater community evenness, except in butterflies. However, there are distinct dynamics of recovery among groups. For example, mine tailings did not affect butterfly β‐diversity, but caused extirpation of dominant species of ants, dung beetles, and bees. Moreover, ants and dung beetles followed a divergent trajectory characterized by higher turnover, which enhanced spatial differentiation. In contrast, bee communities were shaped by the persistence of widespread taxa, which ultimately influenced regional homogenization. These divergent responses can be explained by two mechanisms potentially associated with community‐wide species traits: (i) because the deposition of tailings is spatially heterogeneous, it generates varying spatial conditions in the substrate, which alters the permeability and accumulation of organic matter in the soil (do Carmo et al. 2017; de Páez et al. 2024). As a result, these distinct environmental conditions promote the differentiation of ants and dung beetles between the impacted areas (Correa et al. 2026; Ribas et al. 2005); (ii) considering the magnitude of the impact and the increased isolation of habitat patches resulting from dam rupture, the dispersal ability of bees could favor homogenization because only species capable of moving between these patches across the degraded matrix can exploit the remaining floral resources (Brosi et al. 2008; Gámez‐Virués et al. 2015). As a result, mine tailings act as a semi‐permeable matrix, isolating some populations and reducing bee β‐diversity. In contrast, the lower richness and abundance of butterflies may reflect stochastic mortality caused by the abrupt burial and destruction of habitats during the passage of the mine tailings. By removing plants along their path, the tailings destroyed butterfly habitats and food and reproductive resources (Brown Jr. and Freitas 2000). Beyond the adult phase, the stochastic impact of mining tailings affected the larval stage of the butterflies by burying plants containing eggs and eliminating the larvae's food source, thereby compromising future recruitment capacity and drastically slowing the recovery of the local community (Koh et al. 2004).

Additionally, the response of invertebrate communities to dam collapse may indicate cascading changes in soil, mammal and flora communities. The communities of dung beetles, bees, and butterflies may reflect changes in the availability and composition of their key resources (e.g., dung, pollen, and fruits) and sources (e.g., mammals and plants) (Potts et al. 2003; Nichols et al. 2009; Kessler et al. 2011). Changes in dung beetle communities might also reflect the collapse of large‐mammal communities in impacted areas. Declines in herbivorous and omnivorous mammals, which produce large volumes of dung, may have made this resource scarcer and more patchily distributed (Potts et al. 2003; Bogoni et al. 2019). Furthermore, the higher evenness might indicate the loss of specialists due to homogenization of the mammal community into a residual assemblage of generalist or small‐bodied species (Potts et al. 2003; Bogoni et al. 2019). In a similar way, the response to the impact of bee communities might reflect a parallel collapse in floral resources, consistent with the observed impact on plant communities. The removal of dominant plants that exhibit massive flowering events in areas impacted by mining tailings may reduce the availability of nectar and pollen causing a decline in abundance of bees, mainly on dominant species (Nichols et al. 2009; Moroń et al. 2011). We also observed a reduction in plant richness leading to a more homogeneous community, this affects available floral resources and may consequently have caused the loss of specialist bee species (Biesmeijer et al. 2006; Nichols et al. 2009). In contrast to beetles and bees, which often have stricter resource requirements, frugivorous butterflies feed on fermenting resources in the substrate, creating a “bank” of organic matter that accumulates and provides greater stability in food availability (Krenn et al. 2006; Nascimento et al. 2026). Additionally, in the event of fruit scarcity, they exhibit greater dietary plasticity than bees and dung beetles and can replace fruit with feces, carcasses, or plant sap (Krenn et al. 2006). Finally, the response of the epigeic ant community may reveal physical and structural changes in the soil (Ribas et al. 2012). The decline in ant richness and abundance may result from soil compaction and the destruction of nesting microhabitats, leading to the loss of specialized guilds (Philpott et al. 2009; Ribas et al. 2012). These factors, combined with the burial of leaf litter, may have caused a collapse in competitive dominance, favoring an impoverished community dominated by generalist species. Thus, the response of invertebrate communities might provide evidence of collapse in other ecosystem components.

### 4.3 Implications for Environmental Policy and Disaster Management

The Fundão dam collapse represented a major event in the evolution of Brazilian dam‐safety policy and global tailings governance. Together with the subsequent Brumadinho disaster, it contributed to the strengthening of Brazil's National Dam Safety Policy through Federal Law No. 14,066/2020, which expanded requirements related to risk assessment, emergency planning, disaster prevention, monitoring, and tailings‐facility management (Leonhardt et al. 2021). However, the Fundão collapse also exposed an important limitation: the absence of pre‐disturbance ecological baseline data, which constrained our ability to assess the magnitude and persistence of its impacts on biodiversity. The Federal Law No. 14,066/2020 does not explicitly require standardized biodiversity baselines across areas potentially affected by dam failures (Brasil 2020). Although environmental impact assessments are formally required during the licensing process, regulatory agencies often set vague requirements. Although the monitoring conducted in this study represents an important first step toward standardized technical protocols, the scope of biodiversity assessments is still largely shaped by cost‐driven consultancy proposals. Consequently, these assessments often produce generic species inventories rather than functionally informed evaluations capable of supporting long‐term biodiversity monitoring (Dias et al. 2022). Moreover, biodiversity monitoring remains an overwhelmingly reactive tool, almost always triggered by environmental disasters or legal mandates following impacts, rather than established as a proactive baseline tool. Similarly, although the Global Industry Standard on Tailings Management requires baseline knowledge of the environmental context and assessment of the potential ecological consequences of tailings‐facility failures, it provides limited detail on biodiversity‐specific baseline assessments and long‐term monitoring protocols (Global Tailings Review 2020).

The magnitude and persistence of the ecological changes documented here underscore the societal importance of preventing future failures through rigorous dam‐safety standards and risk reduction, while strengthening emergency planning to reduce consequences for downstream communities and biodiversity. Our study therefore emphasizes the need to establish long‐term, spatially replicated biodiversity monitoring around high‐risk mining facilities, encompassing areas potentially exposed to direct and indirect impacts as well as suitable reference sites (see, e.g., Wauchope et al. 2024; Wunder et al. 2025).

## 5 Conclusions and Future Directions

Across taxa, the most consistent pattern was that directly impacted sites experienced strong reductions in abundance, and these abundance changes often reframed α‐diversity patterns once richness was standardized. This indicates that tailings can simultaneously depress population sizes while reorganizing dominance structure (evenness), making inferences based on raw richness alone potentially misleading. The direction and magnitude of responses differed among plants, invertebrates, and vertebrates, consistent with taxa differing in dispersal ability, habitat dependence, trophic position, and sensitivity to substrate and hydrological change. Overall, Fundão tailings generated a mosaic of constraints and opportunities, producing winners and neutral taxa alongside losers rather than a uniform decline across the biota (see, e.g., Neves et al. 2026). Importantly, extirpation was often associated with increased differentiation, indicating that impacted sites did not lose the same species everywhere. Moreover, our results show that the same landscape can exhibit differentiation via site‐specific losses while also showing homogenization via dominance shifts, depending on the taxonomic group and on whether change is evaluated using species identities or abundances.

These findings highlight priorities for both research and management aimed at reducing biodiversity loss and improving recovery after tailings disturbances in the Doce River basin. Future assessments of “biodiversity recovery” after environmental disasters should jointly evaluate three dimensions of biodiversity variation: (i) temporal change in population abundance, (ii) identity turnover (colonization and extirpation), and (iii) dominance structure. Because these dimensions can shift in different directions, integrating them can clarify whether interventions should prioritize rebuilding populations, facilitating recolonization, or preventing dominance‐driven homogenization. More than 10 years after the Fundão collapse, continued investment in long‐term monitoring and taxon‐focused restoration will be essential for targeted strategies that mitigate mining impacts and promote resilient, diverse communities.

## Author Contributions

**Reginaldo A. F. Gusmão:** conceptualization (lead), data curation (lead), formal analysis (lead), investigation (equal), methodology (lead), project administration (lead), visualization (lead), writing – original draft (lead), writing – review and editing (equal). **Eduardo H. Barros:** conceptualization (lead), methodology (equal), project administration (lead), supervision (lead), writing – review and editing (equal). **Tássia J. Bertotto:** data curation (equal), methodology (equal), project administration (equal), writing – review and editing (equal). **Marianne Azevedo‐Silva:** conceptualization (equal), writing – original draft (lead), writing – review and editing (equal). **Tamilie Carvalho:** conceptualization (equal), writing – original draft (lead), writing – review and editing (equal). **Rogerio L. Rodrigues:** conceptualization (equal), data curation (equal), investigation (equal), methodology (equal), project administration (equal), writing – review and editing (equal). **Eduardo B. Segatto:** conceptualization (equal), data curation (equal), investigation (equal), methodology (equal), writing – review and editing (equal). **Renan L. Betzel:** data curation (equal), investigation (equal), methodology (equal), writing – review and editing (equal). **Gustavo P. Pizziolo:** data curation (lead), investigation (equal), methodology (equal), writing – review and editing (equal). **Rafaela G. Gatti:** data curation (equal), methodology (equal), writing – review and editing (equal). **Fernanda J. da Silva:** data curation (equal), methodology (equal), writing – review and editing (equal). **Thiago Gonçalves‐Souza:** conceptualization (lead), data curation (lead), formal analysis (lead), methodology (lead), project administration (equal), validation (lead), visualization (lead), writing – original draft (lead), writing – review and editing (lead).

## Funding

This work was supported by Renova Foundation and Samarco Mineração S.A.

## Conflicts of Interest

The authors declare no conflicts of interest.

## Supporting information


**Figure S1:** Sampling transect design of invertebrates.
**Figure S2:** Sampling plot design of invertebrates.
**Figure S3:** Sampling transect design of vertebrates and plants.
**Figure S4:** Effects of mud tailings on α‐diversity metrics for plants and vertebrates. Model‐based estimates (mean ± 95% CI) of species richness, abundance (number of individuals), and abundance‐standardized richness are shown for plants, anurans, and birds across the impact gradient (REF, iMUD, dMUD) and sampling periods.
**Figure S5:** Effects of mud tailings on α‐diversity metrics for invertebrates. Model‐based estimates (mean ± 95% CI) of species richness, abundance (number of individuals), and abundance‐standardized richness are shown for ants, bees, beetles, butterflies, and dragonflies across the impact gradient (REF, iMUD, dMUD) and sampling periods.
**Figure S6:** Hill‐number α‐diversity profiles for plants and vertebrates across the mud‐impact gradient. Effective number of species (Hill numbers) is shown for ants, bees, beetles, and butterflies in reference (REF), indirectly impacted (iMUD), and directly impacted (dMUD) sites. Profiles are plotted across Hill orders *q* = 0, 1, and 2, where *q* = 0 reflects species richness, *q* = 1 weights species by their relative abundances (the exponential of Shannon diversity), and *q* = 2 emphasizes dominant species (the inverse of Simpson diversity).
**Figure S7:** Hill‐number α‐diversity profiles for invertebrates across the mud‐impact gradient. Effective number of species (Hill numbers) is shown for ants, bees, beetles, and butterflies in reference (REF), indirectly impacted (iMUD), and directly impacted (dMUD) sites. Profiles are plotted across Hill orders *q* = 0, 1, and 2, where *q* = 0 reflects species richness, *q* = 1 weights species by their relative abundances (the exponential of Shannon diversity), and *q* = 2 emphasizes dominant species (the inverse of Simpson diversity).
**Table S5:** Pairwise comparisons of abundance across impact categories. Post hoc contrasts among impact categories (dMUD–iMUD, dMUD–REF, iMUD–REF) were obtained from estimated marginal means of the fitted abundance models using emmeans. Negative estimates indicate lower values in the first impact category listed in the contrast relative to the second.
**Table S6:** Pairwise comparisons of species richness across impact categories. Post hoc contrasts among impact categories (dMUD–iMUD, dMUD–REF, iMUD–REF) were obtained from estimated marginal means of the fitted abundance models using emmeans. Negative estimates indicate lower values in the first impact category listed in the contrast relative to the second.
**Table S7:** Pairwise comparisons of abundance‐standardized richness across impact categories.
**Table S8:** Summary of temporal β‐diversity metrics across taxonomic groups. For each group, we report results for incidence‐ and abundance‐based indices and for total change (Δ*β*Total), colonization‐resultant change (Δ*β*C), and extirpation‐resultant change (Δ*β*E). Values are the average temporal *β* metric with its 95% confidence interval across pairwise comparisons (Pairs). Impact categories are reference (REF), indirect mud impact (iMUD), and direct mud impact (dMUD). Different letters indicate statistically distinguishable groups within each taxonomic group × index type × metric combination.

**Table S1:** Slope and horizontal distance to the mud for studied sites in areas directly impacted by mud (dMUD).

**Table S2:** Slope and horizontal distance to the mud for studied sites in areas indirectly impacted by mud(iMUD).

**Table S3:** Slope and horizontal distance to the mud for studied sites in reference areas (REF).

**Table S4:** Sampling protocol to each taxa group.

## Data Availability

Data openly available in a public repository: https://zenodo.org/records/21761966.
