# Supplementary material for: Species Extirpation and Colonization Reveal Mixed Homogenization and Differentiation Across Terrestrial Taxa After One of the World's Largest Tailings Dam Failures
Source: Ecol Evol. 2026 Aug 19;16(8):e74199. doi: 10.1002/ece3.74199 (PMC13488791; doi:10.1002/ece3.74199)
Supplement: Supplementary file 1 [file ECE3-16-e74199-s003.docx]

**SUPPLEMENTARY MATERIALS**

**Species extirpation and colonization reveal mixed homogenization and differentiation across terrestrial taxa after one of the world’s largest tailings dam failures**

Reginaldo A. F. Gusmão, Eduardo Hoffmam de Barros, Tássia Juliana Bertotto, Marianne Azevedo Silva, Tamilie Carvalho, Rogerio Laurindo Rodrigues, Eduardo Bortolini Segatto, Renan Luxinger Betzel, Gustavo Passos Pizziolo, Rafaela Galina Gatti, Fernanda de Jesus da Silva, and Thiago Gonçalves-Souza

**Supplementary Material 1 - Characteristics of the study area**

**Table S1.** Slope and horizontal distance to the mud for studied sites in areas directly impacted by mud (dMUD).

**Table S2.** Slope and horizontal distance to the mud for studied sites in areas indirectly impacted by mud(iMUD).

**Table S3.** Slope and horizontal distance to the mud for studied sites in reference areas (REF).

**Table S4.** Sampling protocol to each taxa group.

**Supplementary Material 2**

**Figure S1.** Sampling transect design of invertebrates

**Figure S2.** Sampling plot design of invertebrates

**Figure S3.** Sampling transect design of vertebrates and plants

**Supplementary Material 3**

**Table S5.** Pairwise comparisons of abundance across impact categories.

**Table S6.** Pairwise comparisons of species richness across impact categories.

**Table S7.** Pairwise comparisons of abundance-standardized richness across impact categories.

**Table S8.** Summary of temporal β-diversity metrics across taxonomic groups.

**Figure S4.** Effects of mud tailings on α-diversity metrics for plants and vertebrates.

**Figure S5.** Effects of mud tailings on α-diversity metrics for invertebrates.

**Figure S6.** Hill-number α-diversity profiles for plants and vertebrates across the mud-impact gradient.

**Figure S7.** Hill-number α-diversity profiles for invertebrates across the mud-impact gradient.

**Supplementary Material 1**

**Tables S1, S2, S3, and S4 available at** [**https://zenodo.org/records/21761966**](https://zenodo.org/records/21761966)

**Supplementary Material 2**

**Figure S1.**

**Figure S2.**

**Figure S3.**

**Supplementary Material 3**

**Table S5. Pairwise comparisons of abundance across impact categories.** Post hoc contrasts among impact categories (dMUD–iMUD, dMUD–REF, iMUD–REF) were obtained from estimated marginal means of the fitted abundance models using emmeans. Negative estimates indicate lower values in the first impact category listed in the contrast relative to the second.

| **Taxonomic group** | **Contrast** | **Estimate** | **SE** | ***Z*** | ***P*** |
| --- | --- | --- | --- | --- | --- |
| Plants | dMUD - iMUD | -3.02 | 0.12 | -24.798 | <0.001 |
| Plants | dMUD - REF | -2.93 | 0.14 | -20.307 | <0.001 |
| Plants | iMUD - REF | 0.09 | 0.15 | 0.576 | 0.833 |
| Anura | dMUD - iMUD | -1.41 | 0.24 | -5.748 | <0.001 |
| Anura | dMUD - REF | -0.05 | 0.20 | -0.268 | 0.961 |
| Anura | iMUD - REF | 1.35 | 0.30 | 4.553 | <0.001 |
| Birds | dMUD - iMUD | 0.33 | 0.05 | 7.233 | <0.001 |
| Birds | dMUD - REF | 0.28 | 0.06 | 4.986 | <0.001 |
| Birds | iMUD - REF | -0.04 | 0.06 | -0.785 | 0.712 |
| Ants | dMUD - iMUD | -0.77 | 0.05 | -15.833 | <0.001 |
| Ants | dMUD - REF | -1.01 | 0.05 | -19.709 | <0.001 |
| Ants | iMUD - REF | -0.24 | 0.06 | -4.353 | <0.001 |
| Bees | dMUD - iMUD | -1.25 | 0.10 | -13.148 | <0.001 |
| Bees | dMUD - REF | -1.39 | 0.12 | -11.421 | <0.001 |
| Bees | iMUD - REF | -0.13 | 0.13 | -0.989 | 0.584 |
| Beetles | dMUD - iMUD | -1.11 | 0.18 | -6.328 | <0.001 |
| Beetles | dMUD - REF | -1.57 | 0.23 | -6.760 | <0.001 |
| Beetles | iMUD - REF | -0.45 | 0.25 | -1.781 | 0.176 |
| Butterflies | dMUD - iMUD | -0.23 | 0.09 | -2.433 | 0.040 |
| Butterflies | dMUD - REF | -0.28 | 0.12 | -2.302 | 0.056 |
| Butterflies | iMUD - REF | -0.06 | 0.13 | -0.410 | 0.912 |

**Table S6. Pairwise comparisons of species richness across impact categories.** Post hoc contrasts among impact categories (dMUD–iMUD, dMUD–REF, iMUD–REF) were obtained from estimated marginal means of the fitted abundance models using emmeans. Negative estimates indicate lower values in the first impact category listed in the contrast relative to the second.

| **Taxonomic group** | **Contrast** | **Estimate** | **SE** | ***Z*** | ***P*** |
| --- | --- | --- | --- | --- | --- |
| Plants | dMUD - iMUD | -2.379 | 0.077 | -30.817 | 0.000 |
| Plants | dMUD - REF | -2.204 | 0.125 | -17.680 | 0.000 |
| Plants | iMUD - REF | 0.175 | 0.125 | 1.395 | 0.343 |
| Anura | dMUD - iMUD | -0.467 | 0.103 | -4.516 | 0.000 |
| Anura | dMUD - REF | -0.066 | 0.093 | -0.708 | 0.759 |
| Anura | iMUD - REF | 0.401 | 0.122 | 3.298 | 0.003 |
| Birds | dMUD - iMUD | 0.174 | 0.031 | 5.656 | 0.000 |
| Birds | dMUD - REF | 0.150 | 0.041 | 3.692 | 0.001 |
| Birds | iMUD - REF | -0.023 | 0.041 | -0.563 | 0.840 |
| Ants | dMUD - iMUD | -0.480 | 0.042 | -11.537 | 0.000 |
| Ants | dMUD - REF | -0.557 | 0.044 | -12.713 | 0.000 |
| Ants | iMUD - REF | -0.077 | 0.047 | -1.653 | 0.224 |
| Bees | dMUD - iMUD | -0.470 | 0.064 | -7.307 | 0.000 |
| Bees | dMUD - REF | -0.608 | 0.085 | -7.194 | 0.000 |
| Bees | iMUD - REF | -0.138 | 0.091 | -1.518 | 0.282 |
| Beetles | dMUD - iMUD | -0.716 | 0.078 | -9.172 | 0.000 |
| Beetles | dMUD - REF | -0.855 | 0.102 | -8.366 | 0.000 |
| Beetles | iMUD - REF | -0.139 | 0.108 | -1.283 | 0.405 |
| Butterflies | dMUD - iMUD | -0.403 | 0.061 | -6.571 | 0.000 |
| Butterflies | dMUD - REF | -0.561 | 0.077 | -7.322 | 0.000 |
| Butterflies | iMUD - REF | -0.158 | 0.083 | -1.902 | 0.138 |

**Table S7. Pairwise comparisons of abundance-standardized richness across impact categories.** Post hoc contrasts among impact categories (dMUD–iMUD, dMUD–REF, iMUD–REF) were obtained from estimated marginal means (EMMs) of the fitted abundance models using emmeans (impact as the marginal effect). For each taxonomic group, the table reports the contrast estimate, standard error (SE), z-ratio, and associated p-value. Negative estimates indicate lower values in the first impact category listed in the contrast relative to the

| **Taxonomic group** | **Contrast** | **Estimate** | **SE** | ***Z*** | ***P*** |
| --- | --- | --- | --- | --- | --- |
| Plants | dMUD - iMUD | 1.785 | 0.088 | 11.768 | <0.001 |
| Plants | dMUD - REF | 2.034 | 0.177 | 8.181 | <0.001 |
| Plants | iMUD - REF | 1.14 | 0.093 | 1.607 | 0.243 |
| Anura | dMUD - iMUD | 2.518 | 0.333 | 6.97 | <0.001 |
| Anura | dMUD - REF | 1.117 | 0.141 | 0.875 | 0.656 |
| Anura | iMUD - REF | 0.444 | 0.074 | 4.85 | <0.001 |
| Birds | dMUD - iMUD | 0.861 | 0.022 | 5.876 | <0.001 |
| Birds | dMUD - REF | 0.883 | 0.029 | 3.841 | <0.001 |
| Birds | iMUD - REF | 1.025 | 0.034 | 0.753 | 0.732 |
| Ants | dMUD - iMUD | 1.292 | 0.044 | 7.492 | <0.001 |
| Ants | dMUD - REF | 1.522 | 0.05 | 12.737 | <0.001 |
| Ants | iMUD - REF | 1.178 | 0.04 | 4.781 | <0.001 |
| Bees | dMUD - iMUD | 1.862 | 0.125 | 9.284 | <0.001 |
| Bees | dMUD - REF | 1.957 | 0.134 | 9.784 | <0.001 |
| Bees | iMUD - REF | 1.051 | 0.08 | 0.651 | 0.792 |
| Beetles | dMUD - iMUD | 1.501 | 0.177 | 3.441 | 0.002 |
| Beetles | dMUD - REF | 2.242 | 0.309 | 5.866 | <0.001 |
| Beetles | iMUD - REF | 1.494 | 0.222 | 2.707 | 0.019 |
| Butterflies | dMUD - iMUD | 0.847 | 0.049 | 2.904 | 0.01 |
| Butterflies | dMUD - REF | 0.771 | 0.051 | 3.926 | <0.001 |
| Butterflies | iMUD - REF | 0.911 | 0.066 | 1.302 | 0.394 |

**Table S8.** Summary of temporal β-diversity metrics across taxonomic groups. For each group, we report results for incidence- and abundance-based indices and for total change (ΔβTotal), colonisation-resultant change (ΔβC), and extirpation-resultant change (ΔβE). Values are the average temporal β metric with its 95% confidence interval across pairwise comparisons (Pairs). Impact categories are reference (REF), indirect mud impact (iMUD), and direct mud impact (dMUD). Different letters indicate statistically distinguishable groups within each taxonomic group × index type × metric combination.

| **Taxon** | **Index type** | **Metric** | **Impact** | **Pairs** | **Average beta (95 CI)** | **Differences** |
| --- | --- | --- | --- | --- | --- | --- |
| Plants | incidence | ΔβTotal | REF | 633 | 0.0029 (0.0020, 0.0039) | a |
| Plants | incidence | ΔβTotal | iMUD | 722 | 0.0034 (0.0025, 0.0043) | a |
| Plants | incidence | ΔβTotal | dMUD | 2557 | 0.0184 (0.0159, 0.0210) | b |
| Plants | incidence | ΔβC | REF | 633 | 0.0028 (0.0021, 0.0035) | a |
| Plants | incidence | ΔβC | iMUD | 722 | 0.0034 (0.0027, 0.0041) | a |
| Plants | incidence | ΔβC | dMUD | 2557 | 0.0060 (0.0048, 0.0073) | b |
| Plants | incidence | ΔβE | REF | 633 | 0.0001 (-0.0005, 0.0007) | a |
| Plants | incidence | ΔβE | iMUD | 722 | -0.0000 (-0.0007, 0.0006) | a |
| Plants | incidence | ΔβE | dMUD | 2557 | 0.0124 (0.0103, 0.0146) | b |
| Plants | abundance | ΔβTotal | REF | 633 | 0.0003 (-0.0004, 0.0009) | a |
| Plants | abundance | ΔβTotal | iMUD | 722 | 0.0003 (-0.0004, 0.0009) | a |
| Plants | abundance | ΔβTotal | dMUD | 2557 | 0.0135 (0.0104, 0.0167) | b |
| Plants | abundance | ΔβC | REF | 633 | -0.0012 (-0.0016, -0.0007) | a |
| Plants | abundance | ΔβC | iMUD | 722 | -0.0009 (-0.0012, -0.0006) | a |
| Plants | abundance | ΔβC | dMUD | 2557 | 0.0021 (0.0007, 0.0034) | b |
| Plants | abundance | ΔβE | REF | 633 | 0.0014 (0.0010, 0.0019) | a |
| Plants | abundance | ΔβE | iMUD | 722 | 0.0012 (0.0007, 0.0016) | a |
| Plants | abundance | ΔβE | dMUD | 2557 | 0.0115 (0.0089, 0.0141) | b |

| **Taxon** | **Index type** | **Metric** | **Impact** | **Pairs** | **Average beta (95 CI)** | **Differences** |
| --- | --- | --- | --- | --- | --- | --- |
| Ants | incidence | ΔβTotal | REF | 650 | 0.0756 (0.0666, 0.0846) | a |
| Ants | incidence | ΔβTotal | iMUD | 582 | 0.0650 (0.0566, 0.0733) | ab |
| Ants | incidence | ΔβTotal | dMUD | 1656 | 0.0518 (0.0459, 0.0576) | b |
| Ants | incidence | ΔβC | REF | 650 | 0.0800 (0.0714, 0.0885) | a |
| Ants | incidence | ΔβC | iMUD | 582 | 0.0571 (0.0491, 0.0651) | b |
| Ants | incidence | ΔβC | dMUD | 1656 | 0.0390 (0.0332, 0.0447) | c |
| Ants | incidence | ΔβE | REF | 650 | -0.0043 (-0.0063, -0.0024) | a |
| Ants | incidence | ΔβE | iMUD | 582 | 0.0079 (0.0055, 0.0103) | b |
| Ants | incidence | ΔβE | dMUD | 1656 | 0.0128 (0.0116, 0.0140) | c |
| Ants | abundance | ΔβTotal | REF | 650 | 0.0775 (0.0682, 0.0868) | a |
| Ants | abundance | ΔβTotal | iMUD | 582 | 0.0694 (0.0600, 0.0788) | ab |
| Ants | abundance | ΔβTotal | dMUD | 1656 | 0.0575 (0.0501, 0.0649) | b |
| Ants | abundance | ΔβC | REF | 650 | 0.0843 (0.0756, 0.0930) | a |
| Ants | abundance | ΔβC | iMUD | 582 | 0.0657 (0.0567, 0.0748) | b |
| Ants | abundance | ΔβC | dMUD | 1656 | 0.0444 (0.0371, 0.0517) | c |
| Ants | abundance | ΔβE | REF | 650 | -0.0068 (-0.0091, -0.0044) | a |
| Ants | abundance | ΔβE | iMUD | 582 | 0.0036 (0.0008, 0.0065) | b |
| Ants | abundance | ΔβE | dMUD | 1656 | 0.0131 (0.0118, 0.0143) | c |

| **Taxon** | **Index type** | **Metric** | **Impact** | **Pairs** | **Average beta (95 CI)** | **Differences** |
| --- | --- | --- | --- | --- | --- | --- |
| Bees | incidence | ΔβTotal | REF | 564 | -0.0367 (-0.0469, -0.0266) | ab |
| Bees | incidence | ΔβTotal | iMUD | 620 | -0.0265 (-0.0355, -0.0174) | a |
| Bees | incidence | ΔβTotal | dMUD | 1995 | -0.0448 (-0.0511, -0.0385) | b |
| Bees | incidence | ΔβC | REF | 564 | -0.0079 (-0.0180, 0.0021) | a |
| Bees | incidence | ΔβC | iMUD | 620 | 0.0233 (0.0152, 0.0314) | b |
| Bees | incidence | ΔβC | dMUD | 1995 | -0.0431 (-0.0490, -0.0372) | c |
| Bees | incidence | ΔβE | REF | 564 | -0.0288 (-0.0327, -0.0248) | a |
| Bees | incidence | ΔβE | iMUD | 620 | -0.0498 (-0.0546, -0.0450) | b |
| Bees | incidence | ΔβE | dMUD | 1995 | -0.0017 (-0.0039, 0.0005) | c |
| Bees | abundance | ΔβTotal | REF | 564 | -0.0723 (-0.0911, -0.0534) | a |
| Bees | abundance | ΔβTotal | iMUD | 620 | -0.0341 (-0.0509, -0.0174) | b |
| Bees | abundance | ΔβTotal | dMUD | 1995 | -0.0474 (-0.0562, -0.0386) | ab |
| Bees | abundance | ΔβC | REF | 564 | -0.0509 (-0.0686, -0.0332) | a |
| Bees | abundance | ΔβC | iMUD | 620 | -0.0112 (-0.0257, 0.0033) | b |
| Bees | abundance | ΔβC | dMUD | 1995 | -0.0505 (-0.0587, -0.0423) | a |
| Bees | abundance | ΔβE | REF | 564 | -0.0214 (-0.0265, -0.0162) | a |
| Bees | abundance | ΔβE | iMUD | 620 | -0.0229 (-0.0293, -0.0165) | a |
| Bees | abundance | ΔβE | dMUD | 1995 | 0.0031 (0.0012, 0.0050) | b |

| **Taxon** | **Index type** | **Metric** | **Impact** | **Pairs** | **Average beta (95 CI)** | **Differences** |
| --- | --- | --- | --- | --- | --- | --- |
| Beetles | incidence | ΔβTotal | REF | 549 | 0.0032 (-0.0067, 0.0132) | a |
| Beetles | incidence | ΔβTotal | iMUD | 483 | 0.0097 (-0.0012, 0.0206) | a |
| Beetles | incidence | ΔβTotal | dMUD | 1161 | 0.0481 (0.0354, 0.0608) | b |
| Beetles | incidence | ΔβC | REF | 549 | 0.0214 (0.0122, 0.0307) | a |
| Beetles | incidence | ΔβC | iMUD | 483 | 0.0037 (-0.0053, 0.0127) | a |
| Beetles | incidence | ΔβC | dMUD | 1161 | 0.0052 (-0.0061, 0.0165) | a |
| Beetles | incidence | ΔβE | REF | 549 | -0.0182 (-0.0254, -0.0109) | a |
| Beetles | incidence | ΔβE | iMUD | 483 | 0.0060 (-0.0023, 0.0144) | b |
| Beetles | incidence | ΔβE | dMUD | 1161 | 0.0429 (0.0374, 0.0484) | c |
| Beetles | abundance | ΔβTotal | REF | 549 | -0.0248 (-0.0398, -0.0098) | a |
| Beetles | abundance | ΔβTotal | iMUD | 483 | 0.0597 (0.0432, 0.0762) | b |
| Beetles | abundance | ΔβTotal | dMUD | 1175 | 0.0333 (0.0200, 0.0465) | b |
| Beetles | abundance | ΔβC | REF | 549 | -0.0368 (-0.0488, -0.0249) | a |
| Beetles | abundance | ΔβC | iMUD | 483 | 0.0406 (0.0258, 0.0553) | b |
| Beetles | abundance | ΔβC | dMUD | 1175 | 0.0134 (0.0019, 0.0249) | c |
| Beetles | abundance | ΔβE | REF | 549 | 0.0120 (0.0053, 0.0188) | a |
| Beetles | abundance | ΔβE | iMUD | 483 | 0.0192 (0.0129, 0.0254) | a |
| Beetles | abundance | ΔβE | dMUD | 1175 | 0.0199 (0.0152, 0.0245) | a |

| **Taxon** | **Index type** | **Metric** | **Impact** | **Pairs** | **Average beta (95 CI)** | **Differences** |
| --- | --- | --- | --- | --- | --- | --- |
| Butterflies | incidence | ΔβTotal | REF | 571 | -0.0258 (-0.0373, -0.0144) | a |
| Butterflies | incidence | ΔβTotal | iMUD | 545 | -0.0236 (-0.0343, -0.0129) | a |
| Butterflies | incidence | ΔβTotal | dMUD | 2309 | 0.0118 (0.0060, 0.0177) | b |
| Butterflies | incidence | ΔβC | REF | 571 | -0.0511 (-0.0605, -0.0417) | a |
| Butterflies | incidence | ΔβC | iMUD | 545 | -0.0473 (-0.0563, -0.0383) | a |
| Butterflies | incidence | ΔβC | dMUD | 2309 | 0.0164 (0.0108, 0.0220) | b |
| Butterflies | incidence | ΔβE | REF | 571 | 0.0253 (0.0205, 0.0301) | a |
| Butterflies | incidence | ΔβE | iMUD | 545 | 0.0237 (0.0191, 0.0282) | a |
| Butterflies | incidence | ΔβE | dMUD | 2309 | -0.0045 (-0.0078, -0.0012) | b |
| Butterflies | abundance | ΔβTotal | REF | 571 | -0.0219 (-0.0346, -0.0092) | a |
| Butterflies | abundance | ΔβTotal | iMUD | 545 | -0.0216 (-0.0357, -0.0075) | a |
| Butterflies | abundance | ΔβTotal | dMUD | 2309 | -0.0206 (-0.0305, -0.0107) | a |
| Butterflies | abundance | ΔβC | REF | 571 | -0.0425 (-0.0535, -0.0315) | a |
| Butterflies | abundance | ΔβC | iMUD | 545 | -0.0352 (-0.0478, -0.0226) | a |
| Butterflies | abundance | ΔβC | dMUD | 2309 | -0.0332 (-0.0420, -0.0244) | a |
| Butterflies | abundance | ΔβE | REF | 571 | 0.0207 (0.0165, 0.0248) | a |
| Butterflies | abundance | ΔβE | iMUD | 545 | 0.0136 (0.0100, 0.0172) | ab |
| Butterflies | abundance | ΔβE | dMUD | 2309 | 0.0126 (0.0095, 0.0156) | b |

| **Taxon** | **Index type** | **Metric** | **Impact** | **Pairs** | **Average beta (95 CI)** | **Differences** |
| --- | --- | --- | --- | --- | --- | --- |
| Anura | incidence | ΔβTotal | REF | 164 | 0.0207 (-0.0109, 0.0522) | a |
| Anura | incidence | ΔβTotal | iMUD | 144 | 0.0234 (-0.0024, 0.0492) | a |
| Anura | incidence | ΔβTotal | dMUD | 3339 | 0.0299 (0.0237, 0.0362) | a |
| Anura | incidence | ΔβC | REF | 164 | -0.0272 (-0.0553, 0.0009) | a |
| Anura | incidence | ΔβC | iMUD | 144 | -0.0167 (-0.0382, 0.0048) | a |
| Anura | incidence | ΔβC | dMUD | 3339 | -0.0069 (-0.0127, -0.0012) | a |
| Anura | incidence | ΔβE | REF | 164 | 0.0479 (0.0338, 0.0620) | a |
| Anura | incidence | ΔβE | iMUD | 144 | 0.0401 (0.0246, 0.0555) | a |
| Anura | incidence | ΔβE | dMUD | 3339 | 0.0369 (0.0336, 0.0401) | a |
| Anura | abundance | ΔβTotal | REF | 164 | 0.0162 (-0.0157, 0.0481) | a |
| Anura | abundance | ΔβTotal | iMUD | 144 | 0.0323 (0.0022, 0.0625) | a |
| Anura | abundance | ΔβTotal | dMUD | 3347 | 0.0009 (-0.0062, 0.0081) | a |
| Anura | abundance | ΔβC | REF | 164 | -0.0178 (-0.0455, 0.0099) | a |
| Anura | abundance | ΔβC | iMUD | 144 | 0.0027 (-0.0212, 0.0266) | a |
| Anura | abundance | ΔβC | dMUD | 3347 | -0.0135 (-0.0198, -0.0073) | a |
| Anura | abundance | ΔβE | REF | 164 | 0.0340 (0.0214, 0.0465) | a |
| Anura | abundance | ΔβE | iMUD | 144 | 0.0296 (0.0171, 0.0421) | ab |
| Anura | abundance | ΔβE | dMUD | 3347 | 0.0145 (0.0114, 0.0175) | b |

| **Taxon** | **Index type** | **Metric** | **Impact** | **Pairs** | **Average beta (95 CI)** | **Differences** |
| --- | --- | --- | --- | --- | --- | --- |
| Birds | incidence | ΔβTotal | REF | 1168 | -0.0154 (-0.0211, -0.0098) | a |
| Birds | incidence | ΔβTotal | iMUD | 1239 | 0.0025 (-0.0026, 0.0076) | b |
| Birds | incidence | ΔβTotal | dMUD | 1176 | -0.0205 (-0.0258, -0.0152) | a |
| Birds | incidence | ΔβC | REF | 1168 | -0.0339 (-0.0387, -0.0291) | a |
| Birds | incidence | ΔβC | iMUD | 1239 | -0.0252 (-0.0294, -0.0209) | a |
| Birds | incidence | ΔβC | dMUD | 1176 | -0.0481 (-0.0526, -0.0436) | b |
| Birds | incidence | ΔβE | REF | 1168 | 0.0184 (0.0162, 0.0206) | a |
| Birds | incidence | ΔβE | iMUD | 1239 | 0.0277 (0.0258, 0.0296) | b |
| Birds | incidence | ΔβE | dMUD | 1176 | 0.0276 (0.0256, 0.0295) | b |
| Birds | abundance | ΔβTotal | REF | 1168 | -0.0201 (-0.0267, -0.0135) | a |
| Birds | abundance | ΔβTotal | iMUD | 1239 | -0.0016 (-0.0079, 0.0046) | b |
| Birds | abundance | ΔβTotal | dMUD | 1176 | -0.0204 (-0.0267, -0.0141) | a |
| Birds | abundance | ΔβC | REF | 1168 | -0.0291 (-0.0349, -0.0234) | ab |
| Birds | abundance | ΔβC | iMUD | 1239 | -0.0190 (-0.0243, -0.0136) | a |
| Birds | abundance | ΔβC | dMUD | 1176 | -0.0352 (-0.0407, -0.0298) | b |
| Birds | abundance | ΔβE | REF | 1168 | 0.0090 (0.0072, 0.0109) | a |
| Birds | abundance | ΔβE | iMUD | 1239 | 0.0173 (0.0155, 0.0191) | b |
| Birds | abundance | ΔβE | dMUD | 1176 | 0.0148 (0.0132, 0.0165) | b |

**Figure S4. Effects of mud tailings on α-diversity metrics for plants and vertebrates.** Model-based estimates (mean ± 95% CI) of species richness, abundance (number of individuals), and abundance-standardized richness are shown for plants, anurans, and birds across the impact gradient (REF, iMUD, dMUD) and sampling periods.

**Figure S5. Effects of mud tailings on α-diversity metrics for invertebrates.** Model-based estimates (mean ± 95% CI) of species richness, abundance (number of individuals), and abundance-standardized richness are shown for ants, bees, beetles, butterflies, and dragonflies across the impact gradient (REF, iMUD, dMUD) and sampling periods.

**Figure S6. Hill-number α-diversity profiles for plants and vertebrates across the mud-impact gradient.** Effective number of species (Hill numbers) is shown for ants, bees, beetles, and butterflies in reference (REF), indirectly impacted (iMUD), and directly impacted (dMUD) sites. Profiles are plotted across Hill orders q = 0, 1, and 2, where q=0 reflects species richness, q=1 weights species by their relative abundances (the exponential of Shannon diversity), and q=2 emphasizes dominant species (the inverse of Simpson diversity).

**Figure S7. Hill-number α-diversity profiles for invertebrates across the mud-impact gradient.** Effective number of species (Hill numbers) is shown for ants, bees, beetles, and butterflies in reference (REF), indirectly impacted (iMUD), and directly impacted (dMUD) sites. Profiles are plotted across Hill orders q = 0, 1, and 2, where q=0 reflects species richness, q=1 weights species by their relative abundances (the exponential of Shannon diversity), and q=2 emphasizes dominant species (the inverse of Simpson diversity).
